# Neuromeric Organization of the Microbat Brain: Conserved and Distinct Regional Features

**DOI:** 10.1002/cne.70140

**Published:** 2026-02-17

**Authors:** F. Lucero‐Arteaga, A. Abrego‐Alvarez, M. Clauzure, S. Labegorra, V. Heck, A. I. Portu, M. A. Boeris, M. A. Mondino, B. Ribeiro Do‐Couto, K. Y. Tseng, M. Á. García‐Cabezas, J. L. Ferran

**Affiliations:** ^1^ Consejo Nacional de Investigaciones Científicas y Técnicas (CONICET) Buenos Aires Argentina; ^2^ Centro de Producción de Animales de Experimentación (CePAE), Facultad de Ciencias Veterinarias Universidad Nacional de La Pampa General Pico Argentina; ^3^ Facultad de Ciencias Veterinarias Universidad Nacional de La Pampa General Pico Argentina; ^4^ Instituto Murciano de Investigación Biosanitaria Pascual Parrilla–IMIB El Palmar Spain; ^5^ Department of Human Anatomy and Psychobiology, Faculty of Psychology University of Murcia Murcia Spain; ^6^ Department of Anatomy and Cell Biology University of Illinois Chicago—College of Medicine Chicago Illinois USA; ^7^ Department of Anatomy, Histology and Neuroscience, School of Medicine Autonomous University of Madrid Madrid Spain; ^8^ Department of Human Anatomy and Psychobiology, Faculty of Medicine University of Murcia Murcia Spain

**Keywords:** Chiroptera. (min.4–max. 6), Microchiroptera, *Myotis*, neuromeres, prosomeres, *Tadarida*

## Abstract

*Myotis myotis* and *Tadarida brasiliensis* are both microbat species belonging to the Vespertilionidae and Molossidae families, respectively. Our goal is to determine if the 85‐million‐year evolutionary divergence between microbats and the Muridae family (mice and rats) has led to significant regional variations in the brain. However, the 34‐million‐year split between *M. myotis* and *T. brasiliensis* serves as in‐group control to contextualize the larger divergence with rodents. Using the prosomeric framework, main brain derivatives from each neuromeric partition were compared between these two microbats and with rodents. We found that although the fundamental neuromeric organization is conserved across microbats (*M. myotis* and *T. brasiliensis*) and rodents (rats, mice, and gerbils), there are significant regional differences within distinct derivatives such that microbats exhibit smaller corpus callosum, isocortex, optic chiasm, and cerebellum when compared to rodents. On the other hand, the overall pattern of tyrosine hydroxylase (TH)‐positive processes and tracts in the basal plate of the diencephalon‐midbrain‐rostral hindbrain in both bats is similar to that found in rodents and primates. However, a key difference was found in the medial habenula (MHb). Although *M. myotis* showed selective TH expression in the MHb, this was absent in *T. brasiliensis*. Collectively, these findings suggest that the 85‐million‐year evolutionary divergence between bats and rodents has led to notable regional variations in brain anatomy, even though their basic modular plan remained the same.

## Graphical abstract

The fundamental neuromeric modular plan is remarkably conserved across bats and rodents. However, there are significant regional differences in the size and organization of specific derivatives (A). In addition, both *Tadarida brasiliensis* and *Myotis myotis* share similar general patterns of TH‐positive processes and tracts in the basal plate of the diencephalo‐midbrain‐rostral hindbrain. Interestingly, a notable molecular variation was observed in the medial habenula (MHb) as revealed by TH‐positive processes (B).

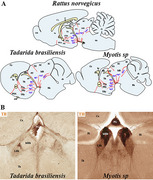

**Abbreviations**
3N: oculomotor complex
3V: third ventricle
A: amygdala
ac: anterior commissure
Acb: accumbens nucleus
A/B: alar‐basal boundary
AON: anterior olfactory nucleus
AP: amygdala, pallial components
Arc: arcuate nucleus
AS: amygdala, subpallial components A/B
At: acroterminal
CA1: field CA1 of the hippocampus
CA2: field CA2 of the hippocampus
CA3: field CA3 of the hippocampus
Cb: cerebellum
cc: corpus callosum
Cd: caudate nucleus (striatum)
cf: cephalic flexure
Cl: claustrum
Co: cochlear nucleus
cp: cerebral peduncle
CPu: caudate‐putamen nucleus (striatum)
Ct: caudal terminal
Cx: cortex
DG: dentate gyrus
dp1: diencephalic prosomere 1
dp2: diencephalic prosomere 2
dp3: diencephalic prosomere 3
dscp: decussation of the superior cerebellar peduncle
ec: external capsule
Ent: entorhinal cortex
fi: fimbria of the hippocampus
fmi: forceps minor of the corpus callosum
fx: fornix
GP: globus pallidus
HB: hindbrain
Hb: habenula
hc: hippocampal commissure
Hi: hippocampus
hp1: hypothalamo‐telencephalic prosomere 1
hp2: hypothalamo‐telencephalic prosomere 2
ic: internal capsule
IC: inferior colliculus
icp: inferior cerebellar peduncle
IP: interpeduncular nucleus
LC: locus coeruleus
LHb: lateral habenula
lot: lateral olfactory tract
M: mammillary body
MB: midbrain
MBbp: midbrain basal plate
mcp: middle cerebellar peduncle
Me: medullary domain (Medulla Oblongata)
ME: medial eminence
MHb: medial habenula
mlf: medial longitudinal fascicle
mp1: midbrain prosomere 1
mp2: midbrain prosomere 2
mth: mammillothalamic tract
NH: neurohypophysis
ns: nigrostriatal tract
OB: olfactory bulb
och: optic chiasm
on: optic nerve
ot: optic tract
P: pons/pontine domain
Pa: paraventricular nucleus
PAG: periaqueductal gray
pc: posterior commissure
pf: pontine flexure
PG: pineal gland
PHy: peduncular hypothalamus
Pir: piriform cortex
Pn: pontine nuclei
POA: preoptic area
PrP: prepontine domain
pt: pyramidal tract
PT: pretectum
PTh: prethalamus
Pu: putamen nucleus (striatum)
r0: rhombomere 0
r10: rhombomere 10
r11: rhombomere 11
r1c: rhombomere 1 caudal
r1r: rhombomere 1 rostral
r2: rhombomere 2
r3: rhombomere 3
r4: rhombomere 4
r5: rhombomere 5
r6: rhombomere 6
r7: rhombomere 7
r8: rhombomere 8
r9: rhombomere 9
rf: retroflex fasciculus (tract)
Rh: rhombencephalon
RMC: red magnocellular nucleus
RP: retropontine domain
RPC: red parvocellular nucleus
SC: superior colliculus
SCh: suprachiasmatic nucleus
SCo: spinal cord
scp: superior cerebellar peduncle
Se: septum
sm: stria medullaris
SN: substance nigra
SNc: substance nigra, compact
SO: superior olive complex
st: stria terminalis
TG: tectal gray
Th: thalamus
THy: terminal hypothalamus
Tu: olfactory tubercle
VTA: ventral tegmental area
vta‐c: ventral tegmental area cortical tract
vta‐l: ventral tegmental area limbic tract

## 1 Introduction

**FIGURE 1:**
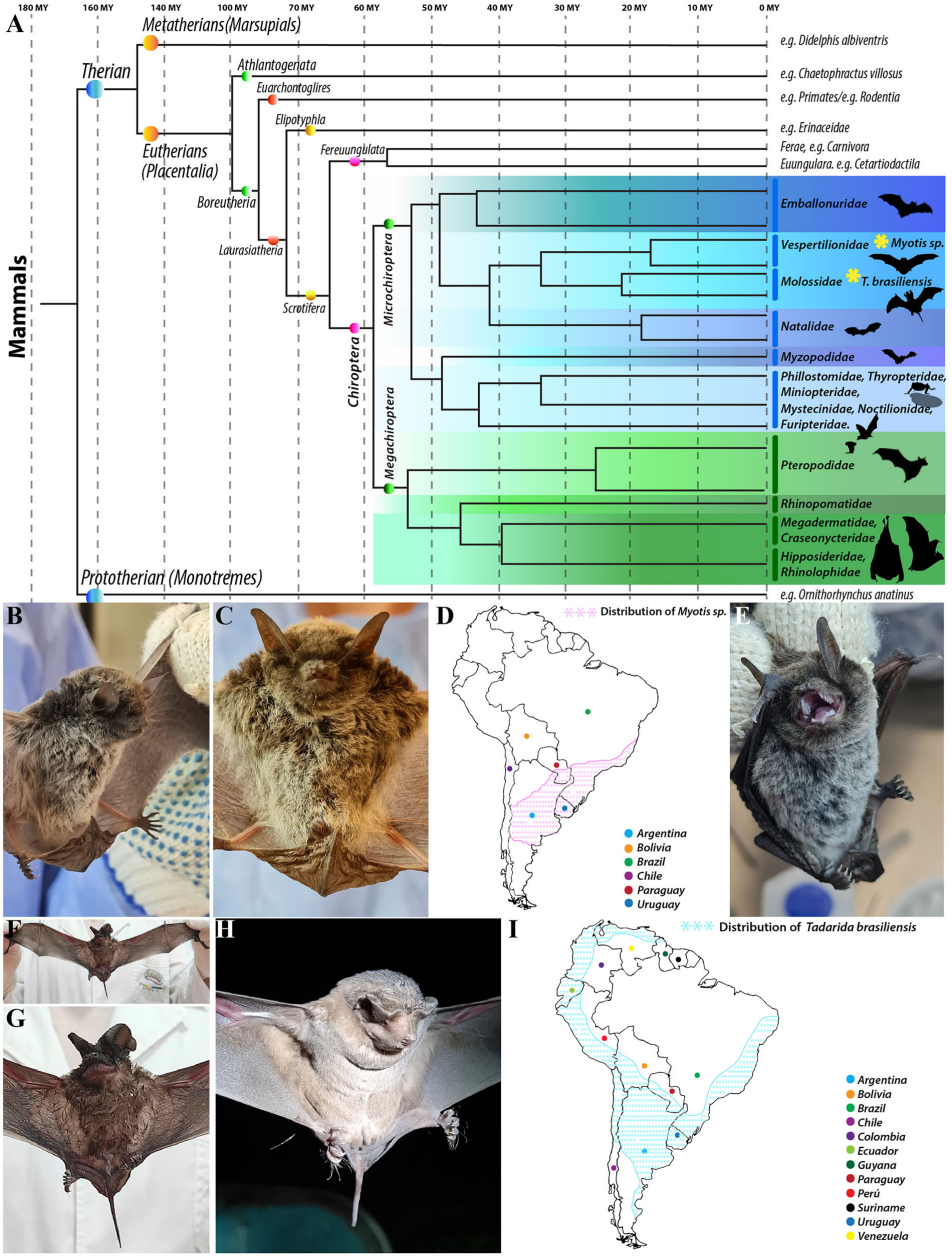
(A) This simplified phylogenetic tree of mammals illustrates the evolutionary relationships among some families of microbats (Microchiroptera) and macrobats (Megachiroptera) within the order Chiroptera, an order that emerged from a common ancestor around 57 million years ago. The two highlighted species (yellow asterisk) under study are a *Myotis myotis bat* (family Vespertilionidae, known as mouse‐eared bat or evening bats) and a *Tadarida brasiliensis* bat (family Molossidae, known as free‐tailed bats). The common ancestor between *M. myotis* and *T. brasiliensis* can be placed around 34 million years ago. For a broader perspective, the common ancestor of the superorders Laurasiatheria (including bats) and Euarchontoglires (including primates and rodents) existed about 85 million years ago (Delsuc et al. 2016; Halliday et al. 2019; Prevosti et al. 2021; Foley et al. 2023). (B–E) The *M. myotis*, or mouse‐eared bat, is a diverse group within the Vespertilionidae family, with approximately 137 species. Although they are primarily insectivorous (feeding on arthropods), some species are also frugivorous. This genus has a specific distribution, with several species found in various South American countries (D). (F–I) The free‐tailed bat (*T. brasiliensis*) is a highly social, insectivorous species in the family Molossidae. It is widely distributed across North, Central, and South America (I).

*Myotis myotis* (*M. myotis*) and *Tadarida brasiliensis* (*T. brasiliensis*) are both extant species within the Microchiroptera suborder, and part of the larger Chiroptera order that emerged from a common ancestor around 57 million years (Mys) ago, subsequently diverging into Megachiroptera (megabat) and Microchiroptera (microbat). This evolutionary timeline is, however, considerably more recent than the estimated 85 Mys of origin of the shared common ancestor between Laurasiatheria (which includes bats) and Euarchontoglires (the superorder containing rodents or primates, among others) (Figure 1A) (Delsuc et al. 2016; Foley et al. 2023; Halliday et al. 2019; Prevosti et al. 2021). The genus *Myotis*, which belongs to the Vespertilionidae family, is one of the most widespread and species‐rich genera of mammals (Figure 1A). These mouse‐eared bats, with approximately 137 recognized species, demonstrate one of the most impressive evolutionary radiations among living mammals (Figure 1B–E) (Bonilla et al. 2024; Gamboa Alurralde and Díaz 2019; Larsen et al. 2012). Although most *Myotis* species primarily feed on arthropods, some are also frugivorous (Gamboa Alurralde and Díaz 2019). The free‐tailed bat, *T. brasiliensis* (Geoffroy 1824), is an insect‐eating bat found throughout North, Central, and South America (Figure 1F–I) (Amaral et al. 2023; Rodríguez‐San Pedro and Allendes 2016). It belongs to the family Molossidae and inhabits diverse roosts, from natural caves to man‐made structures (Amaral et al. 2023; Zegarra et al. 2020). These species form colonies of hundreds to millions of individuals, with different colonies having varied foraging and migration habits. Nine subspecies have been identified, including *T. brasiliensis brasiliensis*, which is widespread in South America (Amaral et al. 2023; Schwartz 1955; Speer et al. 2017). The common ancestor of the Vespertilionidae family, which includes the genus *Myotis*, and the Molossidae family, which includes *T. brasiliensis*, is estimated to have diverged in the late Eocene period. This places their last shared common ancestor around 34 Mys ago (Figure 1A) (Delsuc et al. 2016; Foley et al. 2023; Halliday et al. 2019; Prevosti et al. 2021). The distinct evolutionary trajectories of the greater mouse‐eared bat (*M. myotis*) and the Brazilian free‐tailed bat (*T. brasiliensis*) have resulted in fundamentally different ecological adaptations driven by contrasting wing morphology. *M. myotis* exhibits a flight style that is slow and highly maneuverable, optimized for navigating tight spaces and a specialized foraging niche as a cluttered‐space gleaning insectivore. This adaptation allows it to hunt close to the ground, forest borders, or within open woodland, with a diet consisting primarily of ground‐dwelling insects like beetles and spiders, which are captured by gleaning them from surfaces. Its echolocation strategy involves low‐intensity, high‐frequency calls, which are suitable for detecting the fine echoes from small objects and navigating complex environments characterized by high acoustic clutter (Norberg and Rayner 1987; Russo et al. 2007; Schnitzler et al. 2003). Conversely, *T. brasiliensis* is adapted for a fast, enduring, and straight flight style, which facilitates long‐distance travel and migration. This morphology defines its niche as an open‐space, high‐altitude aerial insectivore, where it hunts fast‐flying aerial insects, such as moths, flies, and midges, high above the canopy or water. Accordingly, its echolocation utilizes high‐intensity, lower frequency calls designed for long‐range detection in open air (Norberg and Rayner 1987; Schnitzler et al. 2003). Finally, these ecological demands lead to contrasting roosting requirements: *M. myotis* prefers warmer, secluded spaces like rock crevices or tree hollows, whereas *T. brasiliensis* requires large, structurally simple areas (e.g., caves or massive human‐made structures) to accommodate its characteristically massive, migratory colonies (Amaral et al. 2023; Zegarra et al. 2020). Thus, the goal of the present study is two‐fold. First, we aim to determine if the 85‐My evolutionary divergence between selected Microchiroptera (microbats) and the Muridae family (mice and rats) was sufficient to allow selective pressures (including genetic, ecological, environmental, and social behavior changes) to cause significant regional variations in the brain. Second, acknowledging the notable ecological and social behavior variations between *M. myotis* and *T. brasiliensis*, we seek to determine if the different selective pressures acting over their more recent 34‐My divergence were sufficient to introduce important variations in their brain structures.

In the present study, we applied the prosomeric framework (L. Puelles and Rubenstein 1993, 2003, 2015) to identify the extent to which key characteristic components of the neuromeric modular organization of the central nervous system (J. L. Ferran et al. 2025; Lucero‐Arteaga et al. 2025) are conserved between two close evolutionary species, *M. myotis* and *T. brasiliensis*. Given that this modular organization is conserved throughout brain development (ontogeny) and evolution (phylogeny) (Albuixech‐Crespo et al. 2017; J. L. Ferran et al. 2022, 2025; J. L. Ferran and Puelles 2019; Lucero‐Arteaga et al. 2025), we anticipate to reveal key brain features that would be considered typical from this clade (suborder *Microchiroptera*).

## 2 Materials and Methods

All animal procedures were conducted in compliance with ethical standards and received approval from their respective institutional ethics committees. Specifically, the Advisory Committee for the Care and Use of Experimental Animals at the Faculty of Veterinary Sciences, University of La Pampa, approved the investigations involving *M. myotis* (RRID:NCBITaxon_51298) and *T. brasiliensis* (RRID:NCBITaxon_9438). All procedures involving rats in this study were approved by the Animal Research Ethics Committee (CEEA) of the University of Murcia. The research was carried out in accordance with Spanish regulations (RD 53/2013, Law 32/2007) and European Union directives (86/609/EEC). Additionally, we followed the FORCED guidelines for the housing and care of rodents (Garrigos et al. 2021).

### 2.1 Bats

Adult bats were collected from rural areas of northern La Pampa Province, Argentina, for a field study. The specimens were then transported to a laboratory for analysis. Mist nets were the primary capture method because they are an efficient and low‐impact way to catch animals. Mist nets were placed strategically at the exits of known bat roosts just before sunset. This timing was chosen to capture bats as they emerged for their nightly activity cycle. Each captured bat was carefully removed from the net and immediately placed in a separate cloth bag. This method prevents contact between animals and minimizes stress during handling. During transport to the laboratory, bats were kept in complete darkness to minimize stress and preserve their natural physiological state. All individuals were processed on the same night as their capture to ensure sample integrity and minimize handling time. Processing involved taxonomic identification, biometric measurements, and other procedures, all performed according to approved ethical and handling protocols.

### 2.2 Rodents

We obtained Sprague–Dawley (SD) rats from the animal facilities at the University of Murcia. All rats were weighed and housed in standard cages (50 cm × 35 cm × 35 cm) with a 2–3 cm layer of dry cork bedding. The housing rooms were maintained at a temperature of 22–25°C and a relative humidity of 45%–60%. Rats had ad libitum access to a standard chow diet (ENVIGO, diet 2014, the United States) and filtered water.

### 2.3 Bats and Rats Brain Tissue Processing

Established protocols were used to collect and process brains from *M. myotis* (RRID:NCBITaxon_51298), *T. brasiliensis* (RRID:NCBITaxon_9438), and *Sprague Dawley* (RRID:RRRC_00239) (J. L. Ferran, Ayad, et al. 2015a; Ferran, Ayad, 2015b). Animals were transcardially perfused with saline solution, then with 4% paraformaldehyde (PFA) in 0.1 M phosphate buffer (PB, pH 7.4). After perfusion, brains were extracted and post‐fixed in PFA at 4°C for 16 h. For free‐floating immunohistochemistry, brains were embedded in 4% agarose and sectioned at 100 µm sagittally or transversely using a Leica Vibratome system. The sections were then mounted on SuperFrost Plus slides for immunohistochemical analysis and scanning (J. L. Ferran, Ayad, et al. 2015a; Ferran, Ayad, 2015b). To study cytoarchitecture, myeloarchitecture, and chemoarchitecture, a subset of brains was prepared using Nissl staining (Lucero‐Arteaga et al. 2025) and Gallyas silver myelin staining (Gallyas 1979; Lucero‐Arteaga et al. 2025). These brains first underwent cryoprotection in 30% phosphate‐buffered sucrose. Following cryoprotection, they were sectioned into 50 µm coronal or sagittal slices with a freezing microtome.

### 2.4 Immunohistochemistry

Our immunohistochemistry procedure followed previously published protocols (J. L. Ferran, Ayad, et al. 2015a; Ferran, Ayad, 2015b). First, we inactivated endogenous peroxidases in tissue sections using 0.3% hydrogen peroxide. Next, sections were incubated for 24/48 h at 4°C with the primary antibody, mouse anti‐NeuN (MAB377, Sigma‐Aldrich, RRID:AB_2298772, 1:4000), rabbit anti‐Calbindin (CB38, Swant, RRID:AB_10000340, 1:4000), and rabbit anti‐tyrosine hydroxylase (TH) (NB300‐109, RRID:AB_10077691, Novusbio, Bio‐Techne R&D Systems, Spain; 1:200). This was followed by a 2/24 h incubation with biotinylated secondary antibodies (goat anti‐rabbit IgG (H + L) and goat anti‐mouse IgG (H + L), Vector Laboratories Cat# BA‐1000, RRID:AB_2313606 and Vector Laboratories Cat# BA‐9200, RRID:AB_2336171, 1:200). Subsequently, sections were exposed to a streptavidin‐peroxidase complex (Vector Laboratories Cat# PK4000, RRID:AB_2336818) for 2 h at room temperature. Finally, we visualized peroxidase activity using 0.03% 3,3′‐diaminobenzidine (DAB) (Sigma, St. Louis, MO, the United States) with 0.003% hydrogen peroxide. We confirmed the specificity of the TH and CB primary antibodies for microbats by Western blot, and non‐specific immunostaining was detected in sections lacking the primary antibody. TH, CB, and NeuN staining patterns in microbats were consistent with those observed in other microchiropteran species (Kruger et al. 2010; Maseko and Manger 2007; Stewart et al. 2021). Specificity of the TH antibody in rats was demonstrated in previous studies (Bilbao et al. 2022; J. L. Ferran et al. 2025; Lucero‐Arteaga et al. 2025).

### 2.5 Western Blot in Bats (Antibody Validation)

Total protein extracts (40 µg) from bats were separated by denaturing 12% SDS–PAGE and then transferred to nitrocellulose membranes. A pre‐stained molecular weight (MW) protein marker (K010, Inbio Highway, Tandil, Argentina) was used for size identification. Membranes were blocked with 5% BSA in PBS for 1 h and subsequently incubated for 3 h with primary monoclonal antibodies against Calbindin, or TH (diluted 1:2000 in PBS with 0.05% v/v Tween‐20). After three washes in PBS with 0.05% v/v Tween‐20 (5 min each), membranes were incubated for 1 h with a goat anti‐rabbit IgG antibody coupled to horseradish peroxidase (diluted 1:2500 in PBS with 0.05% v/v Tween‐20). Finally, the membranes were developed using DAB tablets following the manufacturer's instructions (D4418, Sigma). The specificity of the anti‐Calbindin (CB38, Swant antibody) and anti‐TH (NB300‐109, Novusbio, Bio‐Techne R&D Systems, Spain) antibody in bats was validated using 40 µg of total protein from brain tissue lysates. The lysates were separated by SDS–PAGE and subjected to Western blot analysis. A pre‐stained MW protein marker was run on the same gel to identify the correct band size. The anti‐Calbindin antibody and anti‐TH antibody successfully detected a single band at the expected size of ∼28 and ∼60 kDa, respectively, confirming its specificity (Supporting Information section).

### 2.6 Imaging

Tissue sections were digitally scanned using an Aperio Technologies ScanScope CS system (ScanScope CS Aperio Technologies, USA; RRID:SCR_025111) for image acquisition. We then made image adjustments (size, contrast, brightness, and focus) in Adobe Photoshop 2025. Final figures were meticulously assembled using Adobe Illustrator 2025 (Adobe Systems).

### 2.7 Neuromeric Analyses of the Brain

**FIGURE 2:**
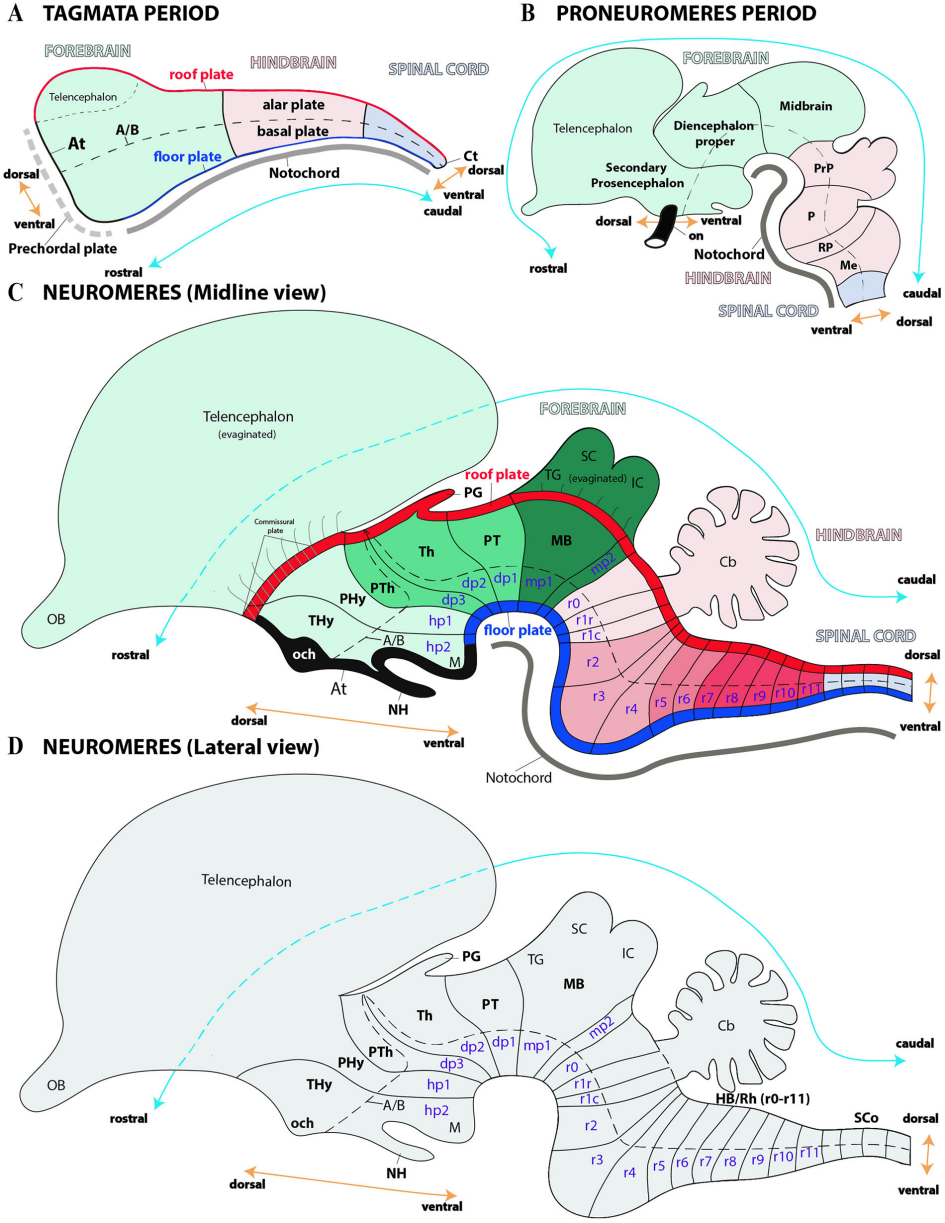
(A–D) Schematic drawings providing a visual timeline of neural tube development, specifically highlighting its regionalization along the anterior‐posterior (AP) and dorsoventral axes (DV). On the basis of the prosomeric framework, these illustrations depict the segmented organization of the vertebrate forebrain—a crucial concept in neurodevelopmental studies. These diagrams show the progressive formation of a complete neuromeric pattern (see below) in sequence: from the early undifferentiated neural tube, through an intermediate stage of initial folding and segmentation, to the final advanced stage with all segments acquired. (A) The early regionalization of the newly closed neural tube establishes its fundamental organization. Along the AP axis, three major compartments—the forebrain (rostral), hindbrain, and spinal cord (caudal)—are formed. These large brain regions, sometimes termed “tagmata,” simultaneously undergo dorsoventral regionalization into four fundamental domains: the roof, alar, basal, and floor plates. Specialized regions, the acroterminal (At) and caudal terminal (Ct) domains, mark where the alar and basal plates converge at the rostral and caudal ends of the neural tube. This process is guided by key inductive signaling centers. The notochord (solid gray line) is a key inductive component that exerts significant influence on the dorsoventral axis, particularly on the development of the floor and basal plates. Similarly, the prechordal plate (dashed gray line), located at the rostral end of the neural tube, is critical for its early regionalization. For clarity of the accompanying schematic representation, the forebrain is pale green, the hindbrain pale pink, and the spinal cord pale blue. The rostro‐caudal (AP) and DV axes are indicated by double pale blue and orange arrows, respectively. (B) During the proneuromeric stage of neural development, the forebrain becomes subdivided into three major AP regions: the secondary prosencephalon (the most rostral), the diencephalon proper, and the midbrain (the most caudal). Concurrently, the hindbrain, or rhombencephalon, is regionalized into four distinct parts that are sequentially arranged from rostral to caudal: the prepontine (PrP), pontine (P), retropontine (RP), and medullary (Me) territories. Notably, the telencephalic territory arises as a dorsal evagination from the alar plate of the secondary prosencephalon. To maintain visual clarity, forebrain derivatives are represented in shades of pale green, hindbrain derivatives in pale pink, and structures related to the spinal cord are shown in pale blue. (C) As development progresses, the segmentation of the brain is completed, and its essential building blocks, the neuromeres, are definitively established. This lateral schematic view of a vertebrate brain highlights midline components, including the roof (red) and floor (blue) plates, to show that neuromeres are composed of these along with the alar and basal plates. This midline schematic helps to identify the telencephalon as an evagination from the alar plate of the secondary prosencephalon, which at this stage is regionalized into two hypothalamo‐prosencephalic prosomeres (hp1/PHy and hp2/THy). Caudally and next to it, the diencephalon proper forms three distinct diencephalic prosomeres (dp1–dp3), whereas the midbrain (MB) differentiates into two prosomeres (mp1 and mp2). These midbrain derivatives are followed by the hindbrain, which exhibits a complex organization of 13 segments known as rhombomeres (r0–r11). The pineal gland (PG) emerges as a derivative of the diencephalic roof plate, and the OCh and neurohypophysis (NH) are derived from the acroterminal (At) domain. (D) This schematic provides a visual outline of the neuromeric organization in the vertebrate neural tube, detailing the alar and basal plate components associated with every defined neuromere. A/B, alar‐basal boundary; Cb, cerebellum; IC, inferior colliculus; MB, midbrain; OB, olfactory bulb; PG, pineal gland; PHy, peduncular hypothalamus; PT, pretectum; PTh, prethalamus; SC, superior colliculus; SCo, spinal cord; TG, tectal gray; Th, thalamus; THy, terminal hypothalamus. *(refer to the accompanying list for full anatomical abbreviations)*.

According to the prosomeric framework, the early neural tube undergoes a process of regionalization (along both rostrocaudal and dorsoventral axes), which in turn establishes the foundational partitions known as primary tagmata along its rostrocaudal axis (Figure 2A). This intricate patterning is critical for the proper development of the forebrain (prosencephalon), hindbrain (rhombencephalon), and spinal cord (Figure 2B,C) (Albuixech‐Crespo et al. 2017; J. L. Ferran et al. 2022, 2025; J. L. Ferran and Puelles 2019; Lucero‐Arteaga et al. 2025; L. Puelles and Rubenstein 1993, 2003, 2015). The forebrain undergoes further regionalization (along its rostrocaudal axis) into three distinct regions: the secondary prosencephalon, the diencephalon proper, and the midbrain (as illustrated in Figure 2B,C). Similarly, the hindbrain undergoes further regionalization into four rostrocaudal proneuromeres: the prepontine (PrP), pontine (P), retropontine (RP), and medullary (Me) regions (Figure 2B,C). Importantly, these proneuromeres undergo further segmentation along the rostrocaudal axis for the formation of neuromeres (Figure 2C,D). Finally, there is also a dorsoventral regionalization during the initial formation of these neuromeric partitions that is characterized by the development of distinct dorsoventral regions: the roof, alar, basal, and floor plates (Figure 2A–C) (L. Puelles 2018; L. Puelles and Rubenstein 1993, 2003, 2015).

## 3 Results

**FIGURE 3:**
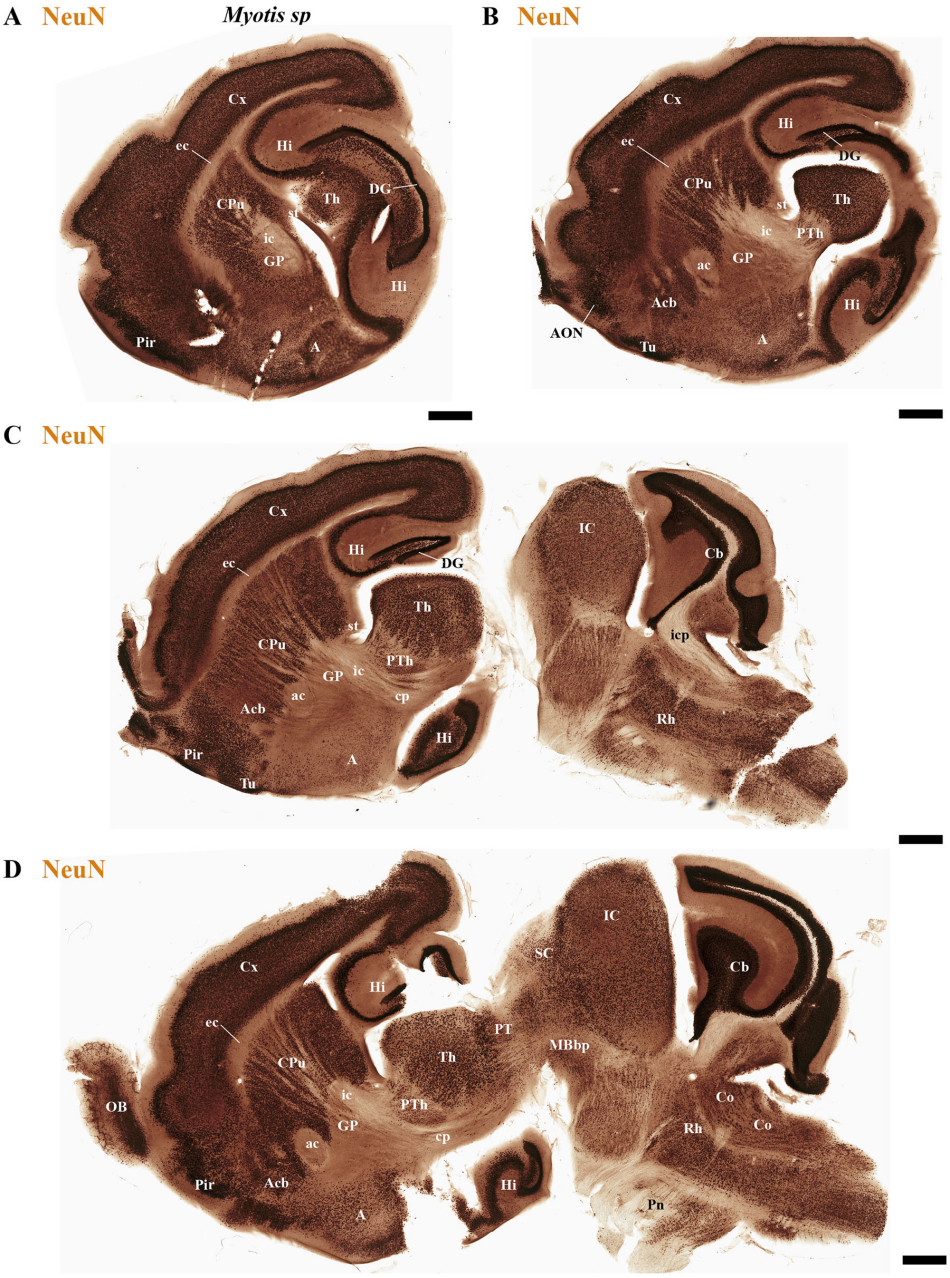
(A–D) Sagittal sections of adult *Myotis myotis*, arranged laterally to medially and stained with NeuN immunohistochemistry, reveal the main derivatives of each neuromeric partition. Prominent features include enlarged anterior commissure (ac) and enlarged inferior colliculus (IC), but also the observation that the expansion of the cerebral cortex (cx) does not cover the midbrain surface. A 500 µm scale bar is applied to all images. *(refer to the accompanying list for full anatomical abbreviations)*.

**FIGURE 4:**
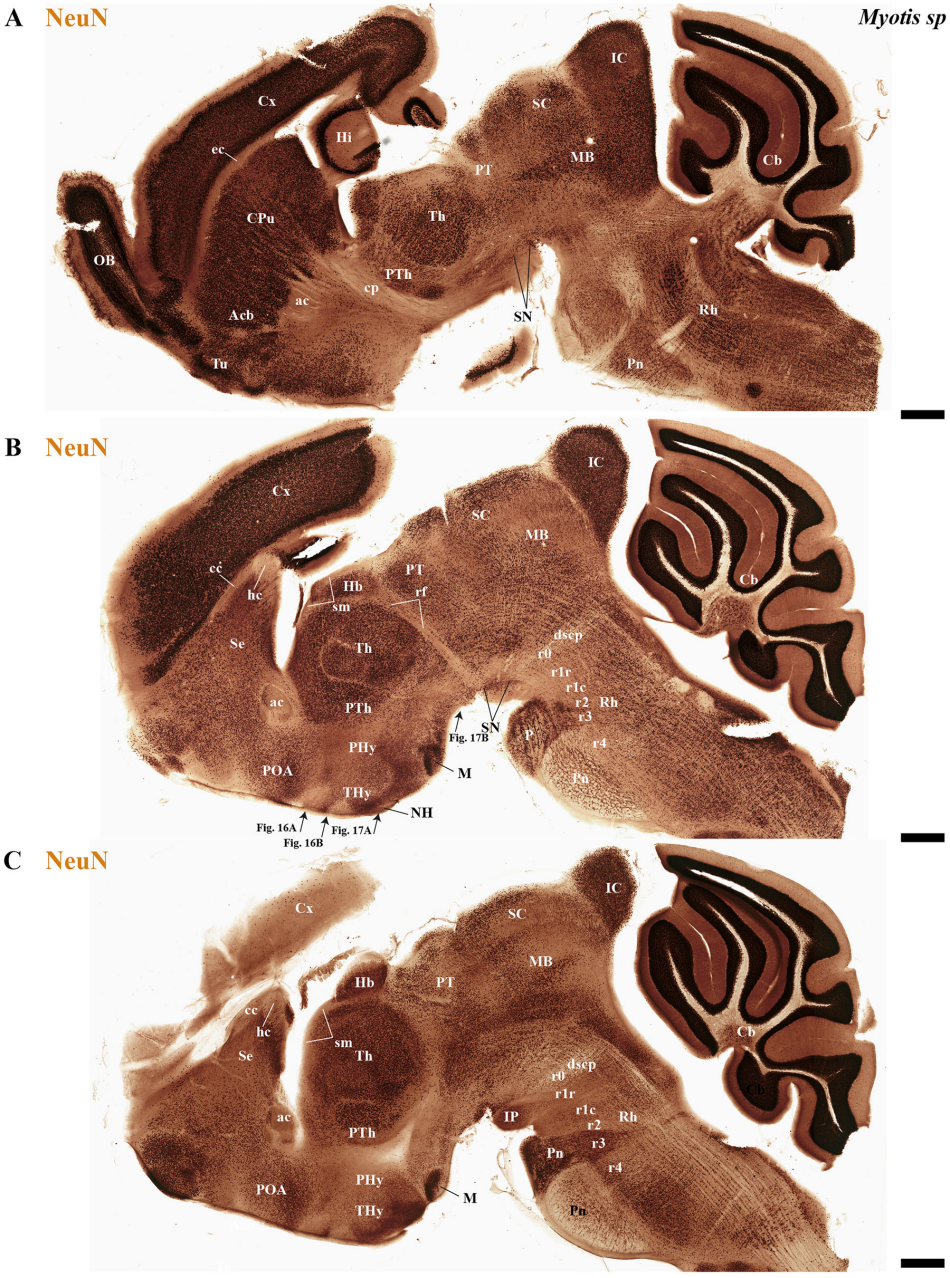
(A–C) A lateral‐to‐medial series of NeuN immunohistochemistry sagittal sections (extended from Figure 3) from adult *Myotis myotis* highlights key neuroanatomical traits: The cerebral cortex (cx) is non‐overlapping with the midbrain (A and B); a reduced corpus callosum (cc) contrasts with a proportionally enlarged hippocampal (hc) and anterior (ac) commissure (B and C); and the hindbrain clearly displays the r0–r4 neuromeres and enlarged pontine nuclei (Pn) (C). The cerebral peduncle (cp) serves to delineate the rostral and caudal extent of the peduncular hypothalamus (PHy). Conversely, the caudal boundary of the retroflex tract (rf) is used to identify the border between dp2 and dp1. A 500 µm scale bar is applied to all images. *(refer to the accompanying list for full anatomical abbreviations)*.

**FIGURE 5:**
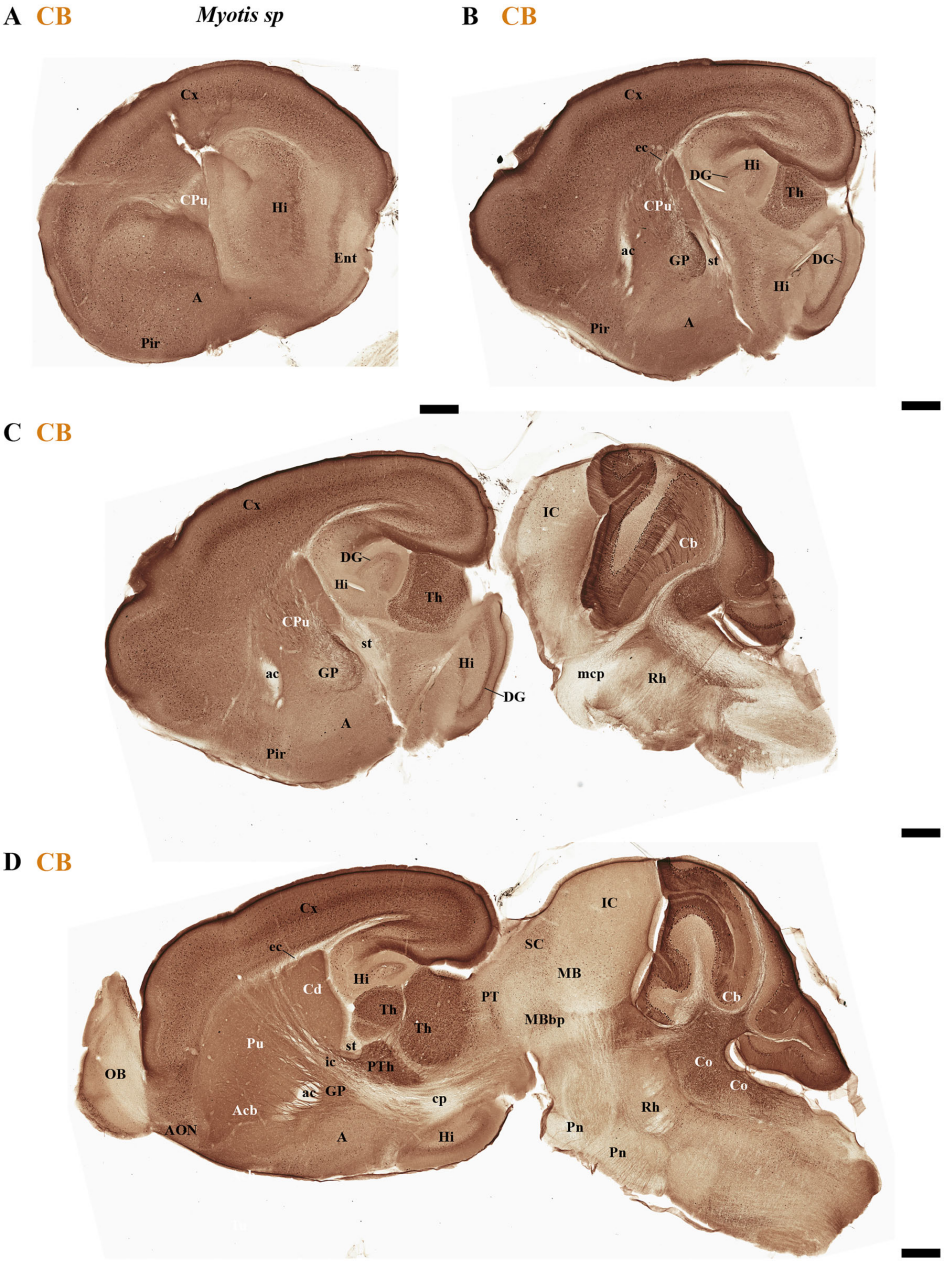
(A–D) Sagittal sections of adult *Myotis myotis*, arranged laterally to medially and stained with Calbindine (CB) immunohistochemistry, that help to recognize mainly thalamic (Th) derivatives but also some prethalamic (PTh) components. As was mentioned in previous figures, prominent anatomical features include enlarged anterior commissure (ac) and enlarged inferior colliculus (IC), and the observation that the expansion of the cerebral cortex (cx) does not cover the midbrain surface. A 500 µm scale bar is applied to all images. *(refer to the accompanying list for full anatomical abbreviations)*.

**FIGURE 6:**
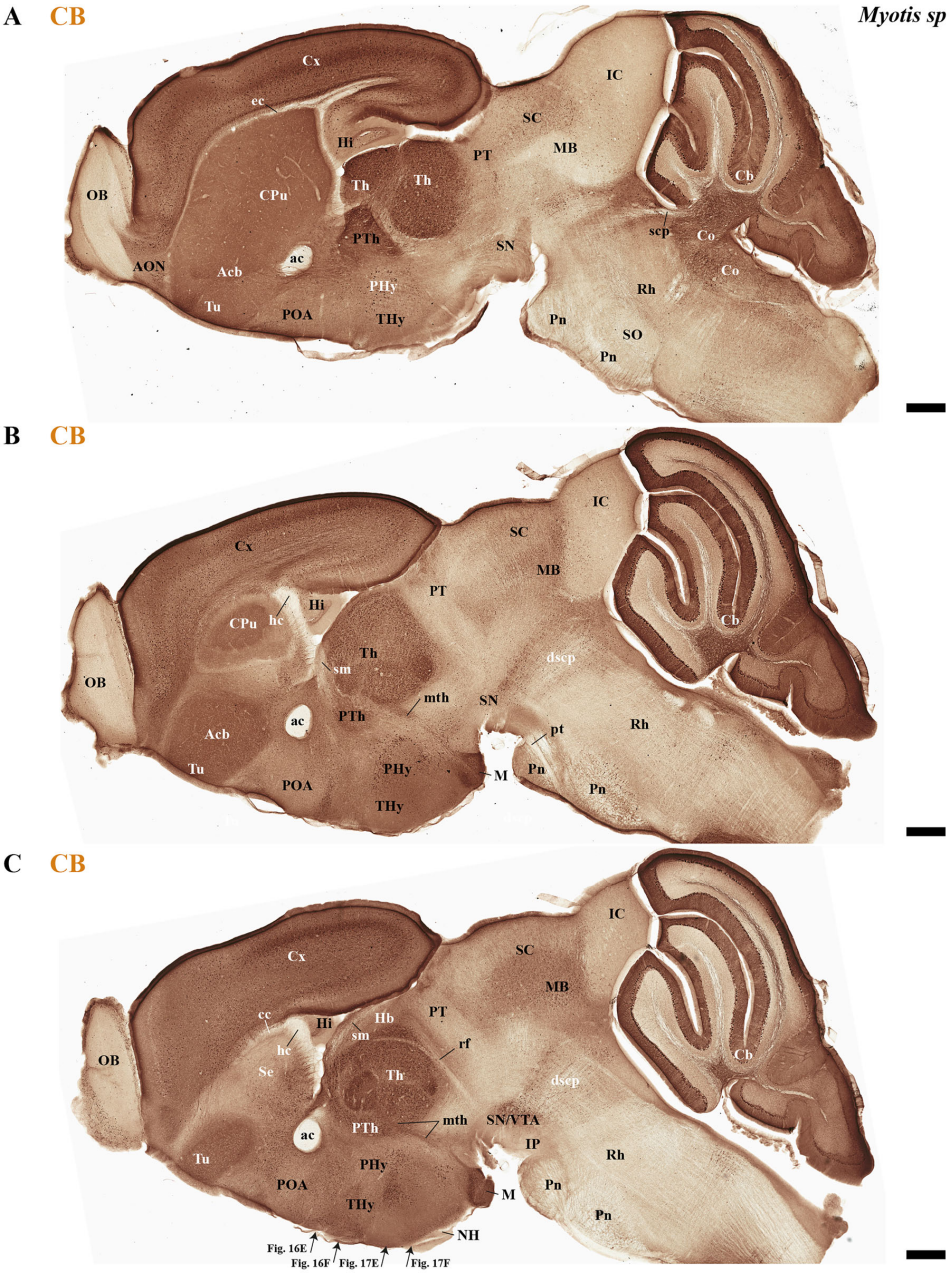
(A–C) A lateral‐to‐medial series of Calbindine (CB) immunohistochemistry sagittal sections (extended from Figure 5) from adult *Myotis myotis* highlights key neuroanatomical traits: The cerebral cortex (cx) is non‐overlapping with the midbrain (A and B); a reduced corpus callosum (cc) contrasts with a proportionally enlarged hippocampal (hc) and anterior (ac) commissure (A–C); and the hindbrain shows enlarged pontine nuclei (Pn) (A–C). The mammillothalamic tract (mth) serves to delineate the border between the dp3 (prethalamus) and dp2 (thalamus) in their alar plate (B and C). Conversely, the caudal boundary of the retroflex tract (rf) is used to identify the border between dp2 and dp1. A 500 µm scale bar is applied to all images. *(refer to the accompanying list for full anatomical abbreviations)*.

**FIGURE 7:**
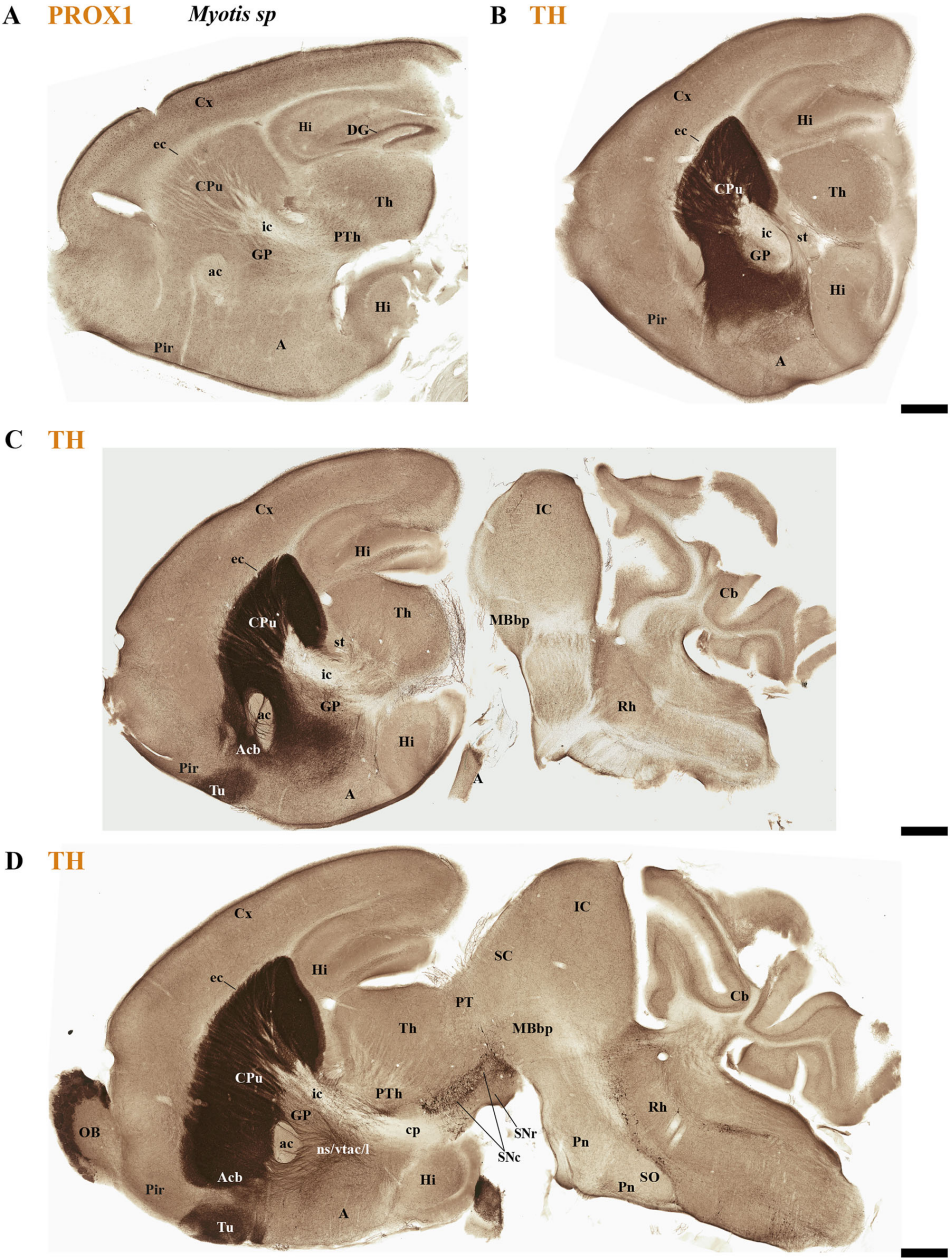
(A) PROX1 immunohistochemistry on a sagittal section from an adult *Myotis myotis* specimen allows for the precise identification and visualization of the dentate gyrus (DG) of the hippocampus. (B–D) A lateral‐to‐medial series of tyrosine hydroxylase (TH)‐stained sagittal sections from adult *M. myotis* reveals TH expression in the caudate‐putamen (CPu), nucleus accumbens (Acb), olfactory tuberculum (Tu), and olfactory bulb (OB) (D). The substantia nigra pars compacta (SNc) and its major efferent tracts—nigrostriatal (ns), VTA‐Acb (vta‐l), and VTA‐prefrontal (vta‐c)—are visible (C and D), with SNc expression mapped to the dp1–dp3, mp1, mp2, and r0 neuromeres (D). Consistent anatomical features include enlarged anterior commissure (ac) and enlarged inferior colliculus (IC), the lack of cerebral cortex (cx) coverage over the midbrain, and the definition of the peduncular hypothalamus prosomere (PHy) boundaries by the cerebral peduncle (cp) (D). A 500 µm scale bar is applied to all images. *(refer to the accompanying list for full anatomical abbreviations)*.

**FIGURE 8:**
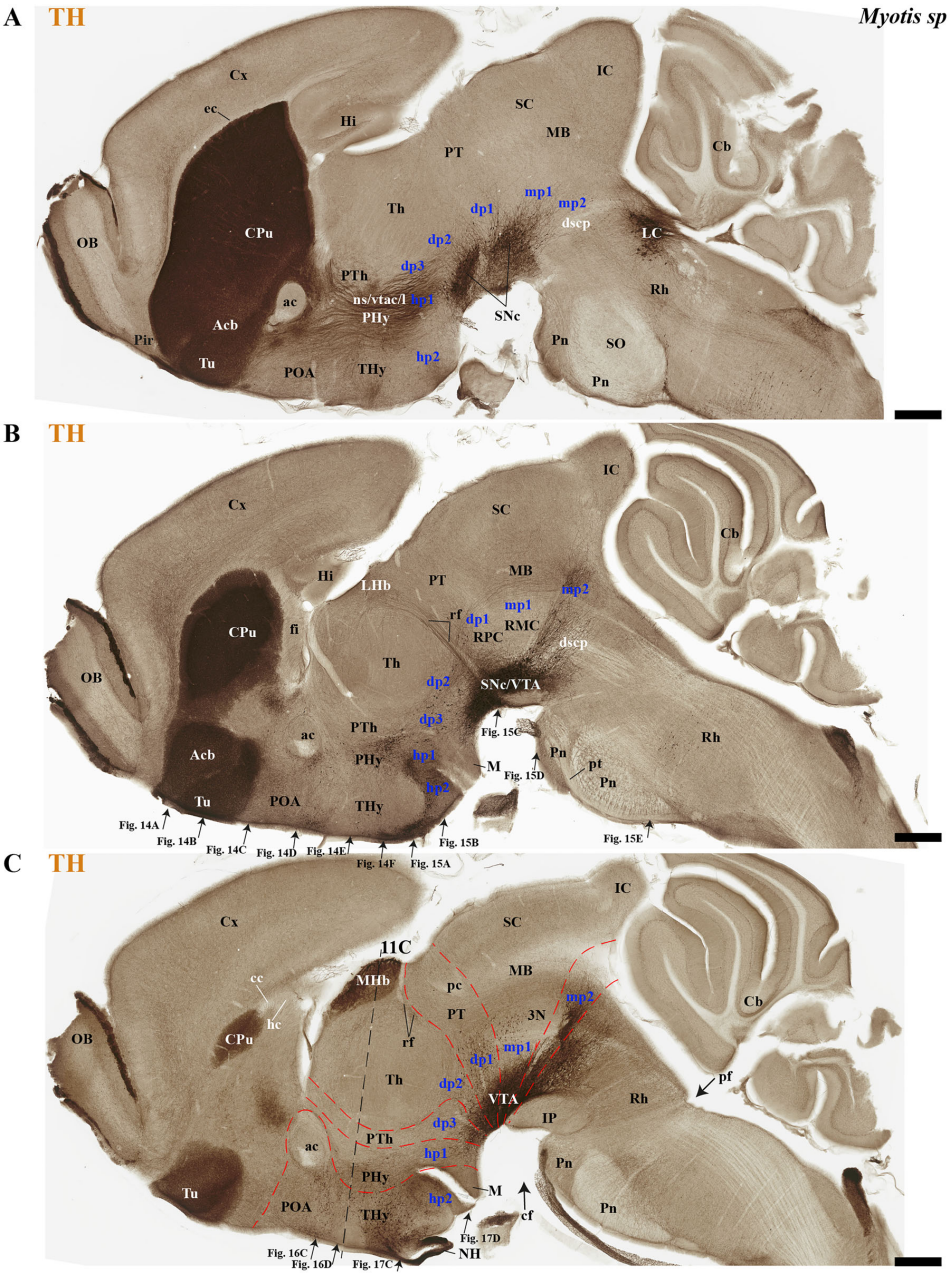
(A–C) A lateral‐to‐medial series of tyrosine hydroxylase (TH) expression in sagittal sections (extended from Figure 7) in adult *Myotis myotis* reveals components of the dopaminergic system and its neuroanatomical context. TH labels striatal derivatives as caudate‐putamen (CPu), nucleus accumbens (Acb), and olfactory tuberculum (Tu) but also the olfactory bulb (OB) (A–C). The substantia nigra pars compacta (SNc) and the ventral tegmental area (VTA) are observed, aligning with the dp1–dp3, mp1, mp2, and r0 neuromeres. Their major efferent tracts—the nigrostriatal (ns), VTA‐Acb (vta‐l), and VTA‐prefrontal (vta‐c)—are also visible (A–C). Noteworthy features include a surface of the cerebral cortex (cx) that is not covering the midbrain (A–C), a reduced corpus callosum (cc) contrasting with enlarged hippocampal (hc) and anterior (ac) commissures (C), and enlarged pontine nuclei (Pn) (A–C). Prosomeres (hp1/2, dp1–dp3, mp1/2) are identified (red lines), with the retroflex tract (rf) marking the dp2–dp1 border (B and C). TH expression is selectively observed in the medial habenula (MHb), contrasting with its absence in the lateral habenula (LHb) (B and C). A 500 µm scale bar is applied to all images. *(refer to the accompanying list for full anatomical abbreviations)*.

**FIGURE 9:**
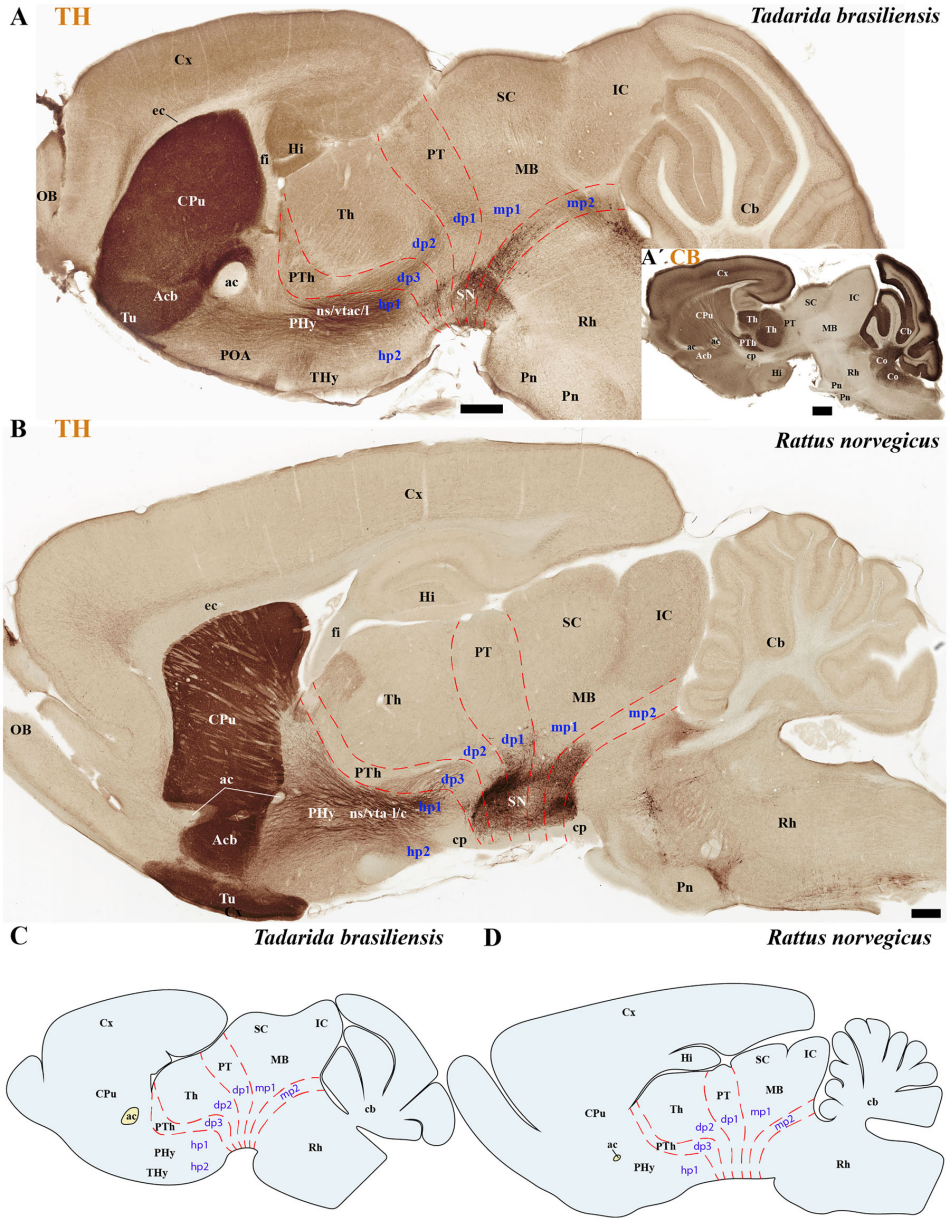
(A–D) A comparison of tyrosine hydroxylase (TH) expression in a selected sagittal brain section of an adult microbat, *Tadarida brasiliensis*, and an adult rodent, *Rattus norvegicus*. (A′) Sagittal section of the *T. brasiliensis* (slightly lateral to the TH) demonstrating Calbindin (CB) expression in PTh and TH derivatives and indicating the location of the cerebral peduncle (cp). This comparison highlights neuroanatomical similarities and differences between these species, which belong to the superorders Laurasiatheria and Euarchontoglires, respectively, and shared a common ancestor approximately 85 million years ago. Microbats, such as *T. brasiliensis* and *M. myotis* (see previous figures), differ from rodents in several neuroanatomical features. Compared to rodents, microbats have a reduced cortex (cx) that does not cover the midbrain surface. Additionally, they exhibit an enlarged anterior commissure (ac), inferior colliculus (IC), and pontine nuclei. The foliation pattern of the microbat cerebellum is also reduced compared to that of rodents. A 500 µm scale bar is applied to all images. *(refer to the accompanying list for full anatomical abbreviations)*.

**FIGURE 10:**
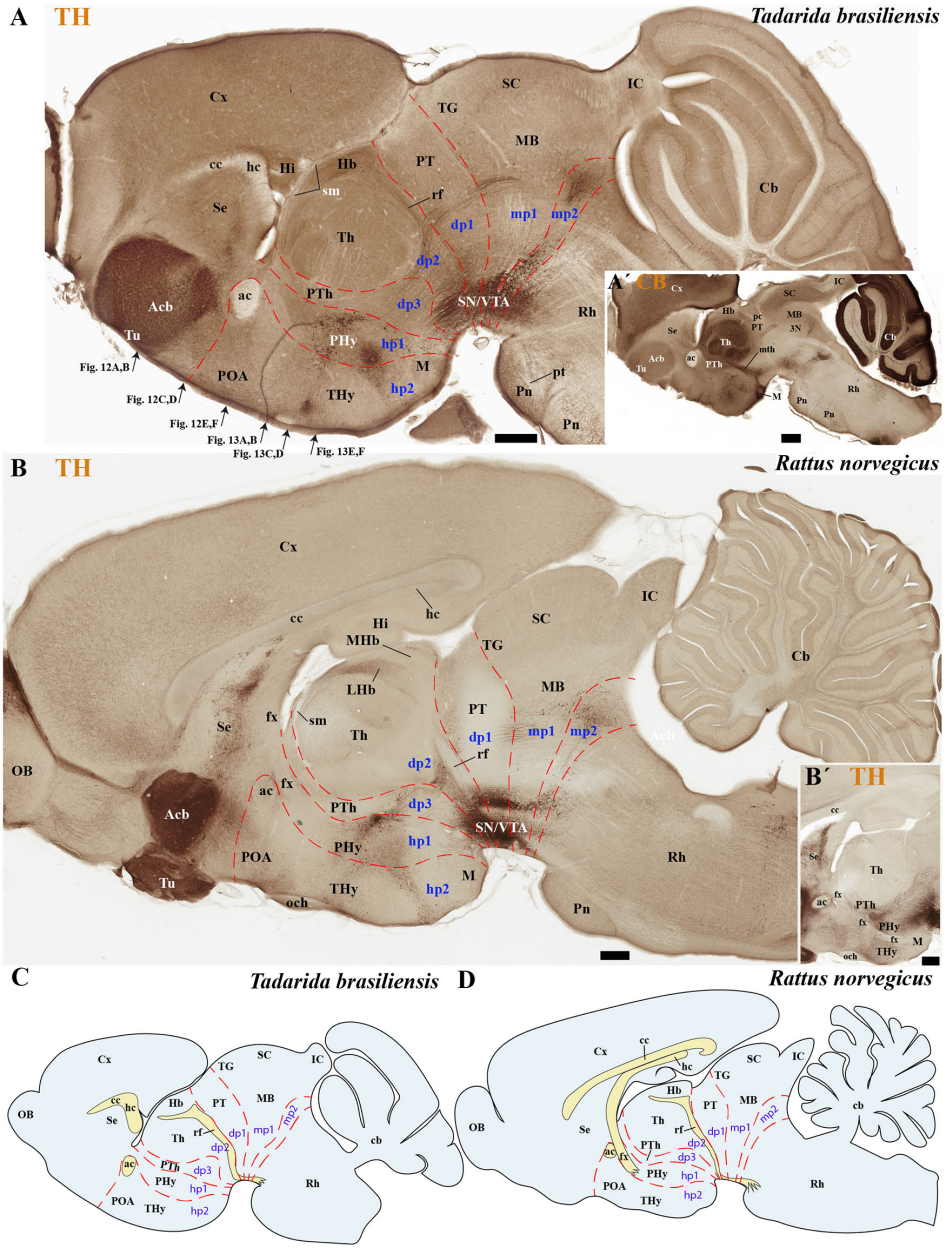
(A–D) This figure compares tyrosine hydroxylase (TH) expression in sagittal brain sections from an adult microbat (*Tadarida brasiliensis*) and an adult rodent (*Rattus norvegicus*). These sections represent a more medial plane of the same specimens shown in Figure 9. (A′) Sagittal section of the *T. brasiliensis* (slightly lateral to the TH) demonstrating Calbindin (CB) expression in PTh and TH derivatives and in the mammillothalamic tract (mth). This comparison highlights neuroanatomical similarities and differences between these species, which belong to the superorders Laurasiatheria and Euarchontoglires, respectively, and shared a common ancestor approximately 85 million years ago. Microbats, such as *T. brasiliensis* and *Myotis myotis* (see previous figures), differ from rodents in several neuroanatomical features. Compared to rodents, microbats have a reduced cortex (cx) that does not cover the midbrain surface. Additionally, they exhibit an enlarged anterior commissure (ac), hippocampal commissure (hc), and pontine nuclei. In contrast, the corpus callosum (cc) and the foliation pattern of the microbat cerebellum are also reduced compared to that of rodents. From this comparison also resulted that a compact postcommissural fornix is absent in microbats (B and B′). A 500 µm scale bar is applied to all images. *(refer to the accompanying list for full anatomical abbreviations)*.

**FIGURE 11:**
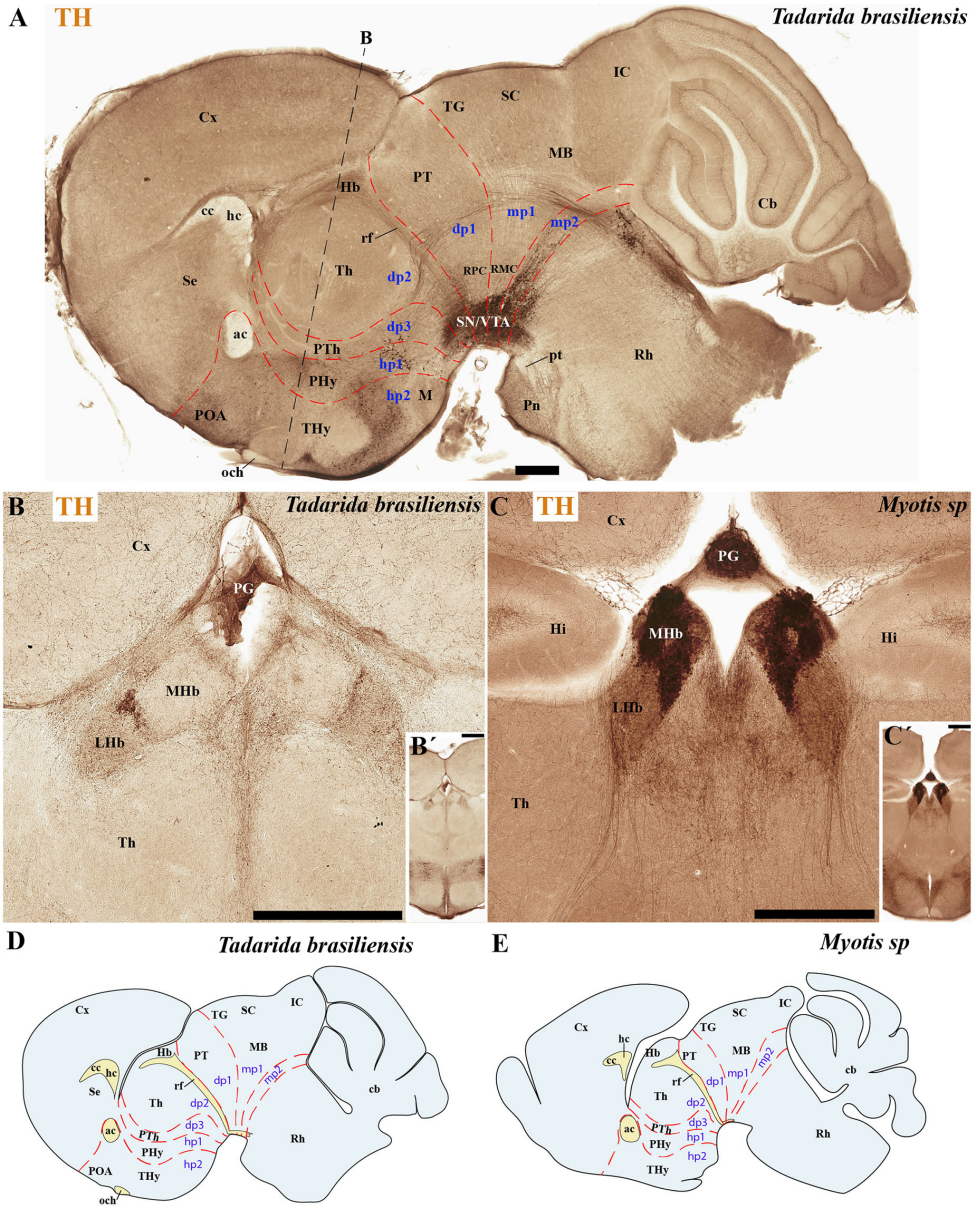
(A–E) Comparative analysis of tyrosine hydroxylase (TH) expression in selected sagittal brain sections from *Tadarida brasiliensis* and *Myotis myotis*, highlighting species‐specific habenular differences. The section from *T. brasiliensis* belongs to the identical specimen featured in Figures 9 and 10; and the section from *M. myotis* belongs to the identical specimen featured in Figures 14 and 15. TH is absent in the lateral habenula (LHb) and medial habenula (MHb) of *T. brasiliensis* (A, B, B′, and D) but only highly expressed in the MHb of *M. myotis* (C, C′, and E). Note the additional high TH expression in the pineal gland (PG) of *M. myotis* (C and C′). Consistent with previous observations in *T. brasiliensis* and *M. myotis*, microbats exhibit several differences when compared to rodents: They possess a reduced cerebral cortex (cx) that does not cover the midbrain surface. Conversely, microbats show an enlarged anterior commissure (ac), hippocampal commissure (hc), and pontine nuclei. In contrast, both the corpus callosum (cc) and the cerebellar foliation pattern are reduced in microbats. The section plane for (B) is indicated in (A); the plane for (C) refers to Figure 8C. A 500 µm scale bar is applied to all images. *(refer to the accompanying list for full anatomical abbreviations)*.

**FIGURE 12:**
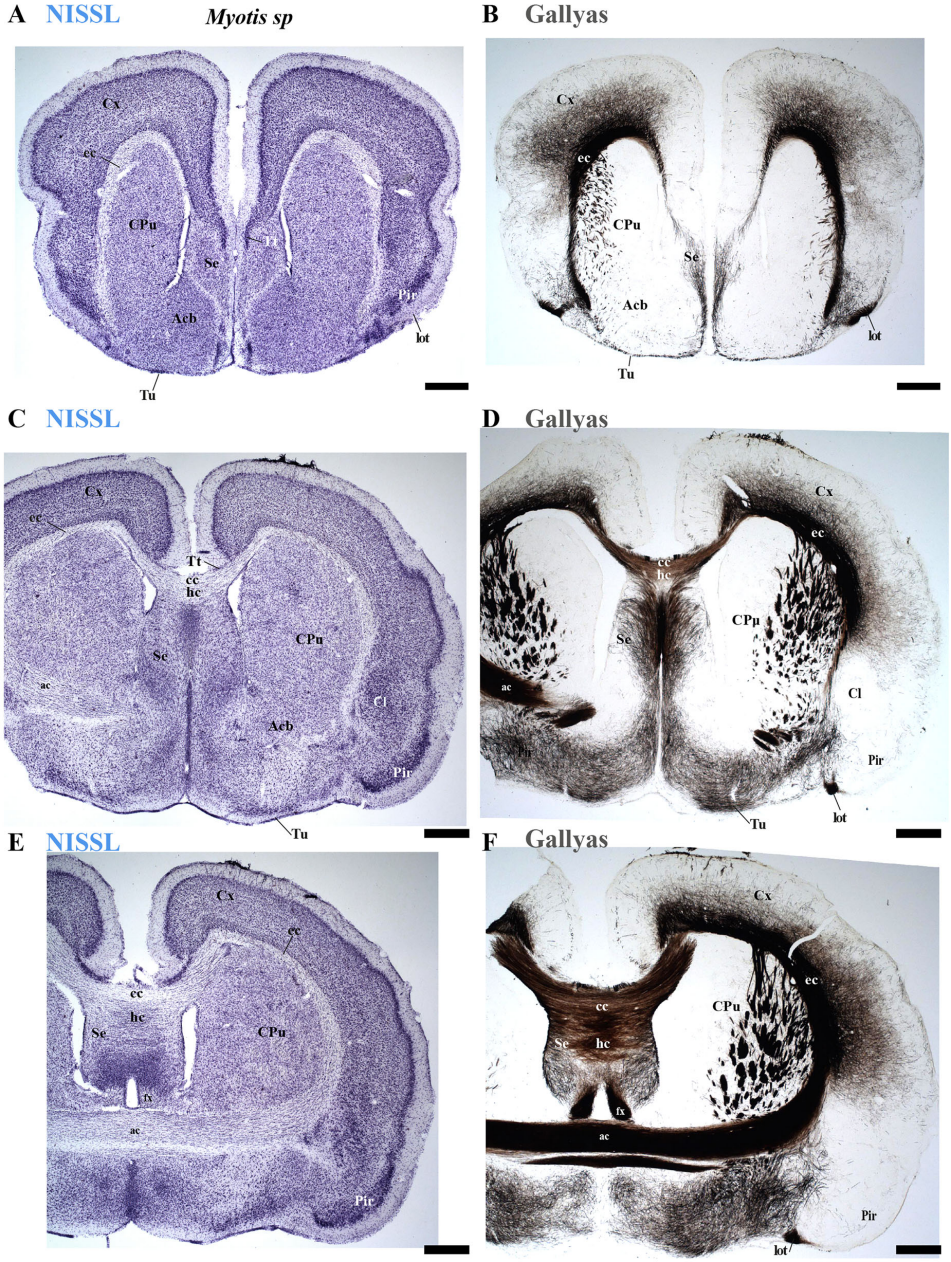
(A–F) A rostrocaudal sequence of consecutive cross sections from an adult *Myotis myotis*, stained with Nissl and Gallyas techniques. The section plane for each image is indicated in Figure 10A. These selected sections display various telencephalic derivatives, including the cortex (cx), caudate‐putamen (CPu), claustrum (Cl), piriform cortex (Pir), septum (Se), and nucleus accumbens (Acb). These structures primarily belong to the peduncular hypothalamus (PHy), though some components (e.g., portions of Se) are derived from the terminal hypothalamus (THy). The images also clearly highlight derivatives of the commissural plate (anterior commissure [ac], corpus callosum [cc], and hippocampal commissure [hc]). The fornix (fx) tract is also observed near the anterior commissure (ac), where its precommissural portion arises. However, the postcommissural part was not identified (E–F). A 500 µm scale bar is applied to all images. *(refer to the accompanying list for full anatomical abbreviations)*.

**FIGURE 13:**
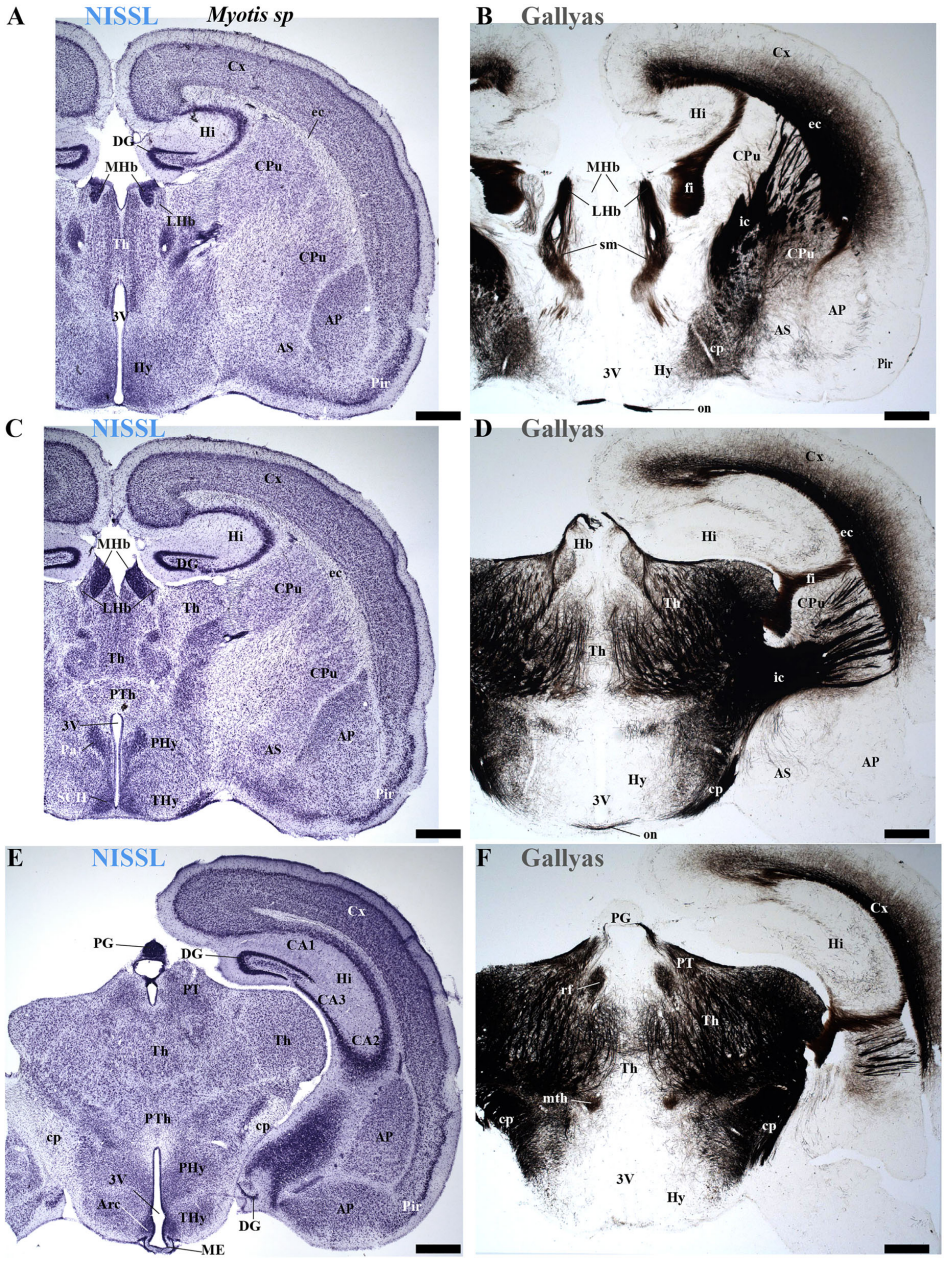
(A–F) A rostrocaudal sequence of consecutive cross sections from an adult *Myotis myotis*, stained with Nissl and Gallyas techniques. This sequence is a continuation of Figure 12, with the plane of section for each image indicated in Figure 10A. Selected sections mainly showing hypothalamic (PHy, THy) and diencephalic (dp1–dp3) derivatives with references to the stria medullaris (sm), mammillothalamic (mth), and retroflex (rf) tracts that help to localize interneuromeric boundaries. Key structures observed in the terminal hypothalamic neuromere (THy) include the suprachiasmatic (SCh) and arcuate (arc) nuclei, along with the median eminence (ME) and optic chiasm (OCh), which are central to the acroterminal region. The paraventricular (Pa) hypothalamic nucleus is indicated within the PHy. Diencephalic structures identified are the prethalamic (dp3), thalamic (dp2), and pretectal (dp1) territories, including the habenular nuclei (MHb, LHb) of the dp2 neuromere. Furthermore, important telencephalic derivatives from the PHy neuromere are visible: caudate‐putamen (CPu), cortex (Cx), hippocampus (Hi), and amygdala (A). The caudal border of the retroflex tract (rf) indicates the dp1–dp2 boundary (F). A 500 µm scale bar is applied to all images. *(refer to the accompanying list for full anatomical abbreviations)*.

**FIGURE 14:**
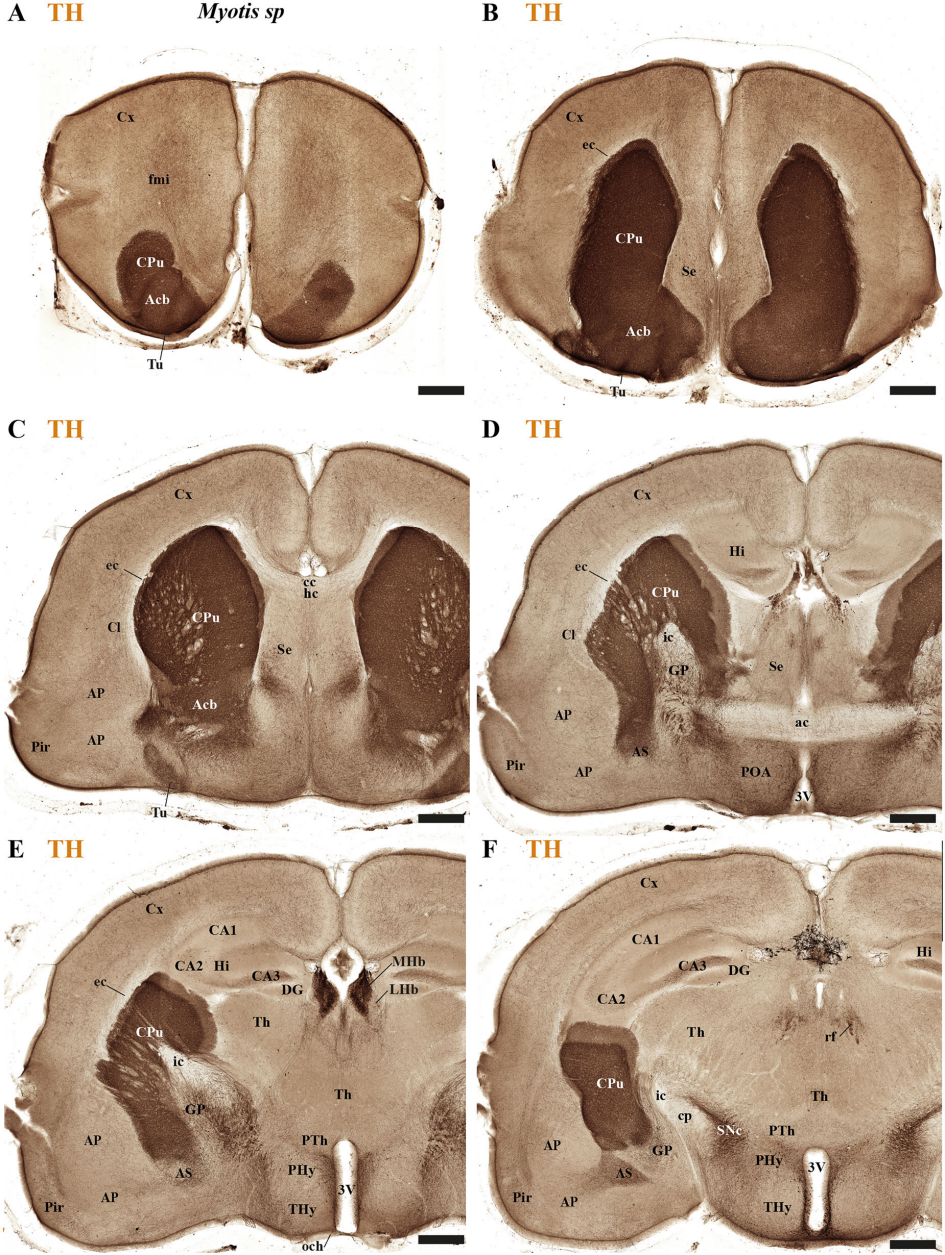
(A–F) A rostrocaudal sequence of consecutive cross sections from an adult *Myotis myotis*, immunostained with tyrosine hydroxylase (TH). The section plane for each image is indicated in Figure 8B. Selected sections illustrate a rostrocaudal sequence of the rostral neural tube neuromeres (prosencephalic), highlighting specific derivatives and anatomical features. TH expression is notably highlighted in the striatal derivatives—specifically the caudate‐putamen (CPu), nucleus accumbens (Acb), and olfactory tuberculum (Tu)—which are identified as part of the peduncular hypothalamus (PHy). A high expression of TH is also observed in the medial habenula (MHb), contrasting sharply with its absence in the adjacent lateral habenula (LHb). The prethalamic (dp3), thalamic (dp2), and pretectal (dp1) regions can be clearly recognized in sections (E and F). Key anatomical observations include the reduced corpus callosum (cc) near the hippocampal commissure (hc) (C) and the enlarged anterior commissure (ac) (D). A reduced optic chiasm is also noted (E). A 500 µm scale bar is applied to all images. *(refer to the accompanying list for full anatomical abbreviations)*.

**FIGURE 15:**
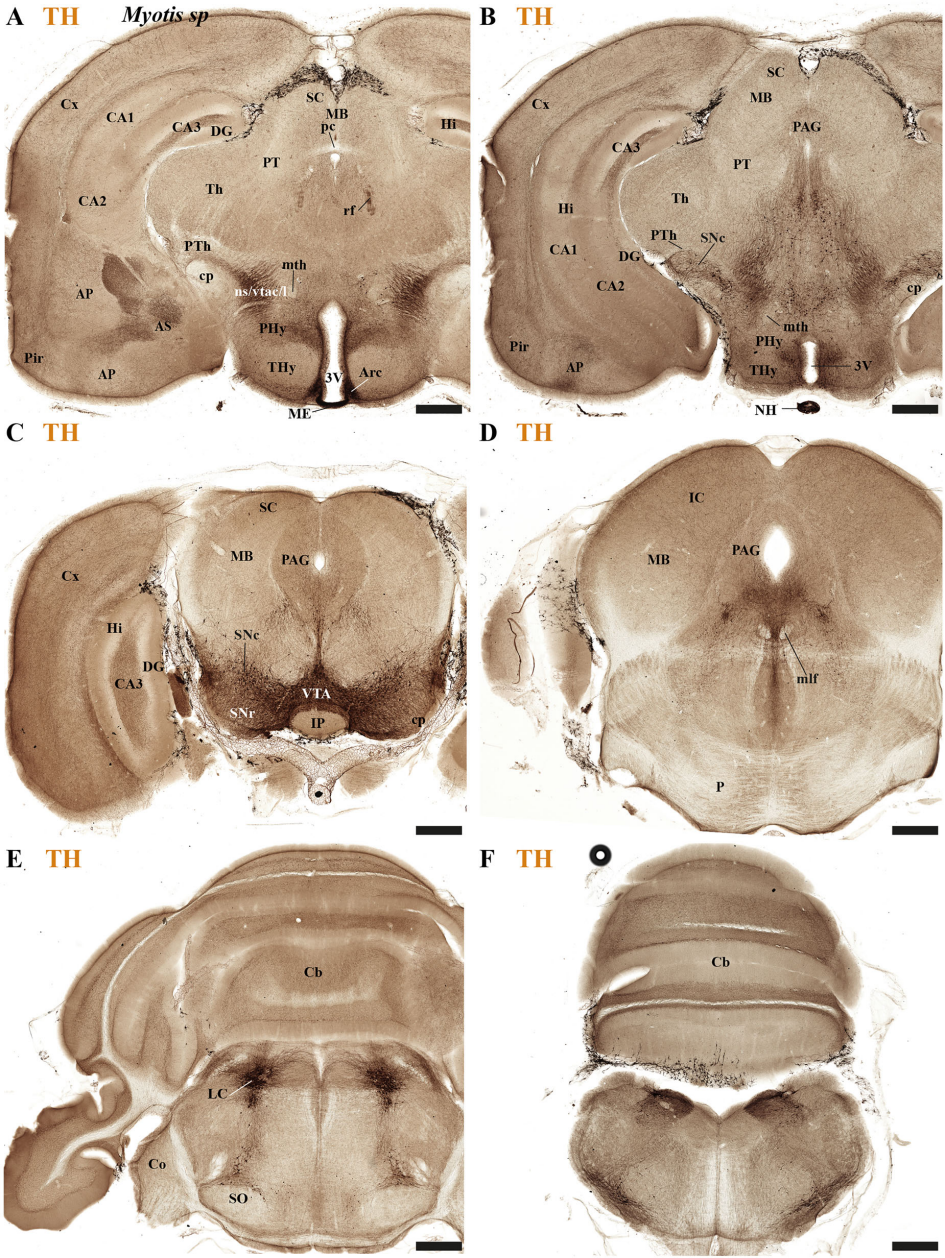
(A–E) A rostrocaudal sequence of consecutive cross sections from an adult *Myotis myotis*, immunostained with tyrosine hydroxylase (TH). This sequence is a continuation of Figure 14, with the plane of section for each image indicated in Figure 8B. (A and B) Selected sections cover a contiguous plane, illustrating the sequence from the terminal (THy) and peduncular (PHy) hypothalamus to the diencephalon proper (prethalamus‐dp3, thalamus‐dp2, pretectal‐dp1), which abuts the midbrain territory (mp1). TH expression is observed in the arcuate nucleus, median eminence, and the substantia nigra compacta (SNc), all derived from the dp3 region. Midbrain (mp1) derivatives, such as the superior and inferior colliculi, are visible in sections (C–F). Section (C) also shows the midbrain's SNc and ventral tegmental area (VTA), along with the adjacent interpeduncular nucleus (IP). Finally, the hindbrain territory is observed, featuring the cerebellar cortex. A 500 µm scale bar is applied to all images. *(refer to the accompanying list for full anatomical abbreviations)*.

**FIGURE 16:**
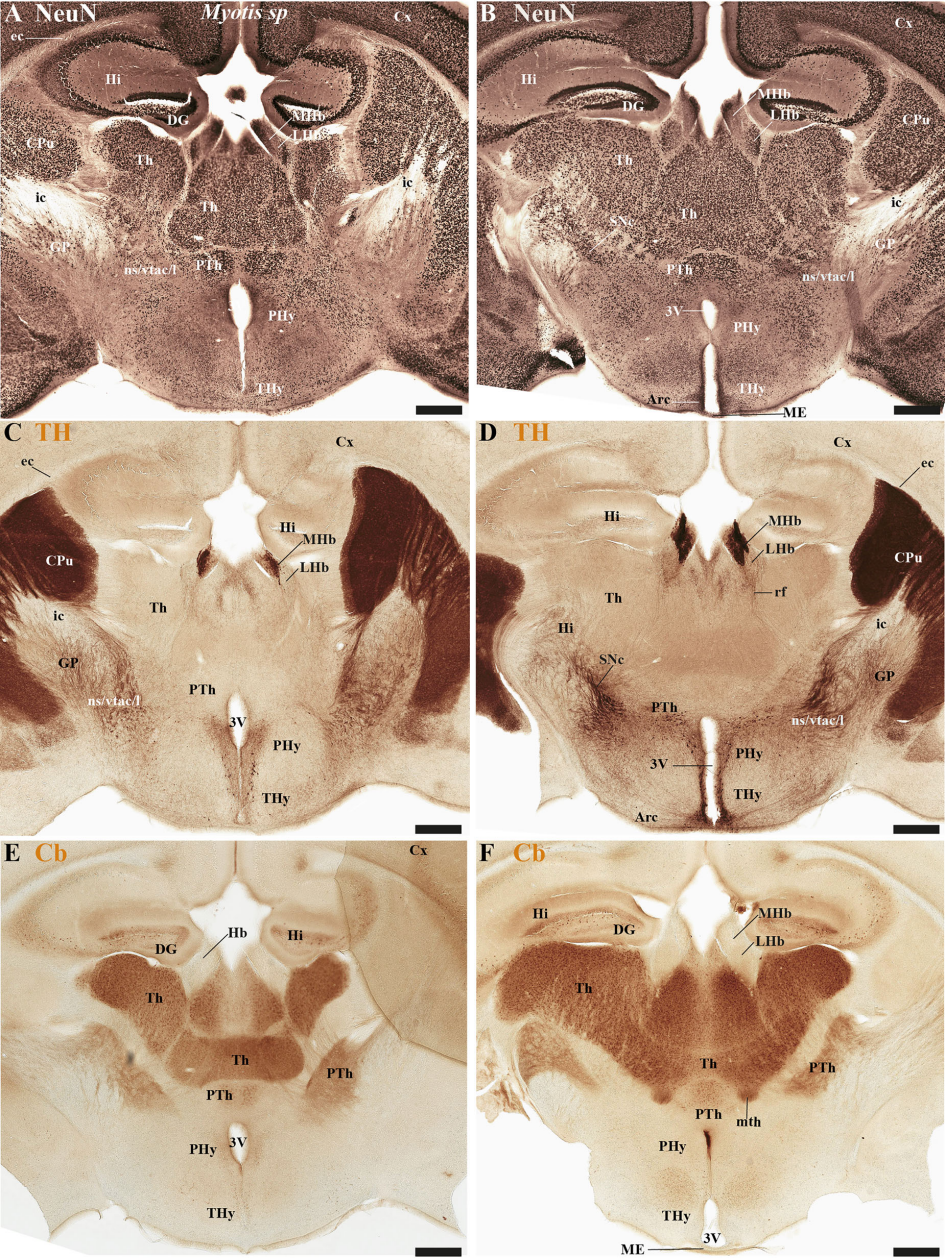
(A–F) A rostrocaudal sequence of consecutive cross sections from an adult *Myotis myotis*, immunostained consecutively with NeuN, tyrosine hydroxylase (TH), and Calbindine (CB). The section plane for each image is indicated in Figure 4B (NeuN), 8C (TH), and 6C (CB). The three markers serve to identify specific derivatives across the terminal and peduncular hypothalamus (THy and PHy), as well as the prethalamic (dp3) and thalamic (dp2) territories. TH expression is used to highlight the arcuate nucleus (from the rostral hypothalamic neuromere), a periventricular area covering the THy and PHy neuromeres, the highly expressing medial habenula (MHb), and striatal derivatives (CPu). In contrast, Calbindin shows strong expression in some prethalamic derivatives (e.g., the reticular nucleus) and most of the thalamic derivatives but is conspicuously avoided in the habenular region. A 500 µm scale bar is applied to all images. *(refer to the accompanying list for full anatomical abbreviations)*.

**FIGURE 17:**
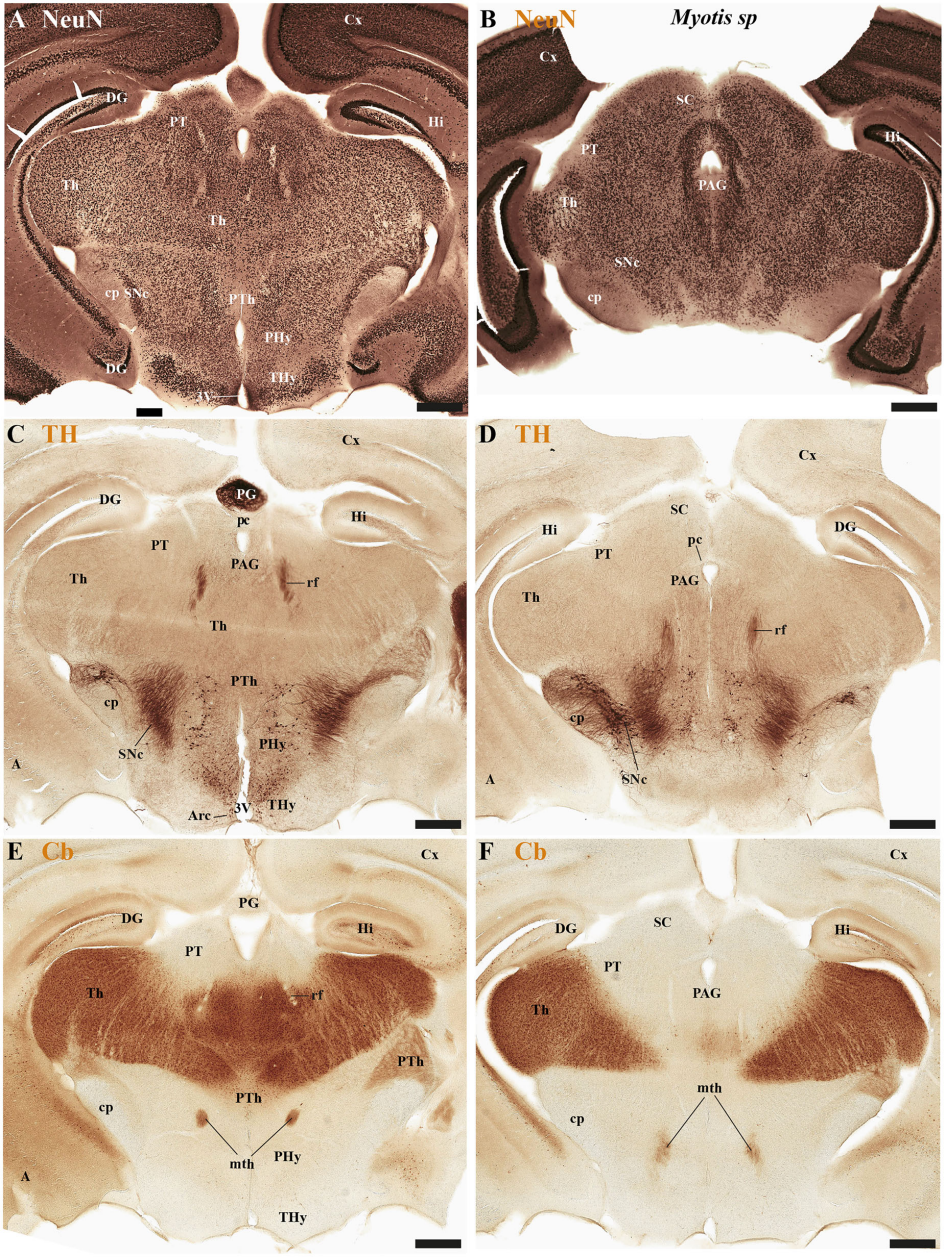
(A–F) A rostrocaudal sequence of consecutive cross sections from an adult *Myotis myotis*, immunostained consecutively with NeuN, tyrosine hydroxylase (TH), and Calbindine (CB). This sequence is a continuation of Figure 16, with the plane of section for each image indicated in Figure 4B (NeuN), 8C (TH), and 6C (CB). The consecutive sequence of sections illustrates the hypothalamic neuromeres (THy, PHy), the diencephalon (prethalamus‐dp3, thalamus‐dp2, pretectum‐dp1), and the rostral midbrain (superior colliculus‐mp1). Calbindin exhibits selective expression in thalamic derivatives, certain prethalamic components, and the mammillothalamic tract. TH expression reveals the substantia nigra compacta (SNc) in two locations: within dp3 (C, near the alar prethalamic components) and within dp2 (D, near the thalamic alar plate derivatives). A 500 µm scale bar is applied to all images. *(refer to the accompanying list for full anatomical abbreviations)*.

This study focused on identifying the neuromeric derivatives and their interneuromeric boundaries in two microchiropteran species: *M. myotis* and *T. brasiliensis*. We identified the chemo‐, cyto‐, and myeloarchitectural features of the neuromeric partitions in the brains of both species. This was accomplished using immunohistochemistry complemented by classical histological techniques (Nissl and Gallyas silver myelin staining) on both sagittal (Figures 3, 4, 5, 6, 7, 8, 9, 10, 11) and transverse sections (Figures 12, 13, 14, 15, 16, 17). We also conducted a detailed analysis of a rat brain for comparison with our findings in microbats (Figures 9B and 10B).

### 3.1 Secondary Prosencephalon of the *M. myotis* and *T. brasiliensis*

At its most rostral end, the neural tube forms the secondary prosencephalon, which is distinctively characterized by two major neuromeric or prosomeric subdivisions, each possessing its own roof, alar, basal, and floor plates. One of them, the peduncular hypothalamo‐telencephalic prosomere (hp1), or peduncular hypothalamus (PHy), is characterized by components of the commissural plate and the tela chorioidea in its roof plate. Its most dorsal alar plate contains both subpallialpa (e.g., diagonal, pallidal, and striatal components, but also subpallial amygdaloid derivatives) and pallial components (e.g., hippocampus, piriform cortex, isocortex, and pallial amygdaloid derivatives). Finally, the hypothalamic components of this neuromere are found in its alar and basal plates. The other neuromere, the terminal hypothalamo‐telencephalic prosomere (hp2), or terminal hypothalamus (THy), presents the anterior commissure (ac) crossing its roof plate, the subpallial preoptic area (POA) in its most dorsal alar plate, and the hypothalamic components located in its alar and basal plates (Figure 1C,D) (Bardet et al. 2010; Bilbao et al. 2022; J. L. Ferran et al. 2025; Ferran, Puelles, 2015; Lucero‐Arteaga et al. 2025; L. Puelles, Martinez‐de‐la‐Torre, Bardet, et al. 2012; L. Puelles and Rubenstein 2015).

The commissural plate spans the roof plate of both the rostral THy, containing the ac, and the caudal PHy, which includes the corpus callosum (cc) and the hippocampal commissure (hc) (also known as the psalterium commissure) (Figure 2C). In *M. myotis* and *T. brasiliensis*, the anterior, cc, and hc were all present (*M. myotis*: Figures 3B–D, 4A–C, 5B–D, 6A–C, 7C–D, 8A–C, 12C–F, and 14C–D; *T. brasiliensis*: Figures 9A, 10A, and 11A). However, compared to rodents, the anterior and hc are proportionally larger, whereas the cc is proportionally smaller (*T. brasiliensis*: Figures 9A–D and 10A–D). The cerebral cortex is also proportionally reduced in both species compared with rodents (see below, *T. brasiliensis*: Figures 9A–D and 10A–D). A crucial tract for identifying the interneuromeric boundary between the PHy and THy in rodents and primates is the fornix tract (J. L. Ferran et al. 2025). Specifically in rodents, the fornix fibers, originating from both hippocampus (fimbria hippocampus), extend to the roof plate. Here, some of them form the body of the fornix, which extends towards the ac, but others cross to the opposite side through the hc. At this point, it divides into a precommissural segment innervating, for example, septal regions and a postcommissural segment going behind the ac and projecting to the mammillary bodies but also to other hypothalamic nuclei (Bilbao et al. 2022; J. L. Ferran et al. 2025; Lucero‐Arteaga et al. 2025). Although the fornix in *M. myotis* and *T. brasiliensis* was readily observed extending from the fimbria hippocampi to the ac, we were unable to observe its compact postcommissural segment, normally seen extending from the posterior border of the ac to the mammillary body (*M. myotis*: Figures 4B,C, 6B,C, 8B,C, 12B,D,F, 13, 16, and 17; *T. brasiliensis*: Figures 10A–D and 11A). Despite the difficulty of localizing the compact postcommissural portion of the fornix, a key reference marker for the PHy and THy, we successfully characterized both neuromeres. This was achieved through the identification of other distinct anatomical landmarks and their derivatives. Keys among these were the nigro‐striatal (ns) tract (linking the substantia nigra to the caudate putamen/striatum), the ventral tegmental area (VTA)‐limbic (vta‐l) tract (projecting from the VTA to the nucleus accumbens [Acb]), and the VTA‐cortical (vta‐c) tract (connecting the VTA to the prefrontal cortex) (J. L. Ferran et al. 2025; Lucero‐Arteaga et al. 2025). These catecholaminergic tracts in *M. myotis* and *T. brasiliensis* were observed to traverse the PHy in a ventrodorsal direction, projecting to their terminal regions: the caudate‐putamen (CPu), nucleus Acb, and prefrontal cortex (*M. myotis*: Figures 7D and 8A; *T. brasiliensis*: Figure 9A). Furthermore, the identification of these tracts aided in defining the rostral PHy‐THy boundary and the caudal PHy‐Dp3 limit. In these microbats, the cerebral peduncle (cp), observed traversing the PHy neuromere, constitutes another critical landmark for differentiating PHy from THy and dp3 (*M. myotis*: Figures 3D, 4A, 5D, and 7D; *T. brasiliensis*: Figure 9A). The cp, a large fiber bundle primarily composed of efferent axons from the cerebral cortex, represents the continuation of the internal capsule (ic). These cortical axons descend through the striatum (CPu), traversing dorsoventrally the PHy neuromere (hypothalamic alar and basal plates). In the PHy alar plate, some of these fibers turn caudally to form the corticothalamic tract. Conversely, fibers continuing through the basal plate turn caudally to constitute the corticobulbar, corticonuclear, corticospinal, and corticoreticular tracts. The cp subsequently forms a prominent longitudinal tract, traversing dp3, dp2, dp1, midbrain prosomere 1 (mp1), midbrain prosomere 2 (mp2), and rostral rhombomeres, ultimately reaching its diverse targets. In the pontine and RP regions, this tract is commonly known as the pyramidal tract (pt), which then differentiates into the lateral and medial corticospinal tracts within the spinal cord. Additionally, ascending thalamocortical tracts originating from the thalamus are incorporated into the alar plate portion of the cp to reach their cortical targets. This path can be observed in both *M. myotis* and *T. brasiliensis* (*M. myotis*: Figures 3B–D, 4A, 5D, 6C, 7A–D, 8B, 13D–F, 14E,F, 15A–C, 16A–D, and 17A–F; *T. brasiliensis*: Figures 10A and 11A).

Within the most rostral neuromere, THy (hp2), well‐known derivatives such as the POA and mammillary bodies are recognizable in both Chiroptera species. Additionally, the most rostral midline components (acroterminal domain) as part of the same neuromere, including the median eminence (ME), arcuate nucleus (Arc), neurohypophysis (NH), and a highly reduced optic chiasm (och), can be identified in both *M. myotis* and *T. brasiliensis* (*M. myotis*: Figures 4B,C, 6A,B,C, 8A,B,C, 13A,C,E, 14D–F, 15A,B, 16A–F, and 17A,C,E; *T. brasiliensis*: Figures 9A, 10A, and 11A).

In *M. myotis* and *T. brasiliensis*, the cerebral cortex, striatum, pallidum, hippocampus, and pallial amygdala—all derivatives of dorsal alar plate of the PHy prosomere—are identified as components of this neuromere. However, a comparison with rodents suggests a proportionally reduced cortex in these Chiroptera species (*T. brasiliensis*: Figures 9A–D and 10A–D). In these microbat species, the cortex only reaches the rostral border of the superior colliculus (SC) and never covers the inferior colliculus (IC) (*M. myotis*: Figures 3D, 4A–C, 5D, 6A–C, 7D, and 8A–C; *T. brasiliensis*: Figures 9A–D, 10A–D, and 11A,D,E). The CPu and the Acb nucleus appear to be proportionally larger in Microchiroptera bats when compared with rodents. Moreover, compared with rodents, the amygdala (mainly its pallial component) appears bigger in *M. myotis* and *T. brasiliensis*, with an apparent increased size in the subpallial and pallial amygdala (*M. myotis*: Figures 3A–D, 5A–D, 7A–D, 13A–D, 14C–F, and 15A,B).

### 3.2 Diencephalon Proper of the *M. myotis* and *T. brasiliensis*

In the chiropteran species selected for study, the diencephalic neuromeres (dp3, dp2, and dp1 prosomeres) were clearly delimited along a rostrocaudal axis (*M. myotis*: Figures 2C,D, 3D, 4A–C, 5D, 6A–C, 7D, and 8A–C; *T. brasiliensis*: Figures 9A, 10A, and 11A). From rostral to caudal, these neuromeres are defined by the prethalamus within the dp3 alar plate, the thalamus and habenular regions in the dp2 alar plate, and the pretectal region in the dp1 alar plate (Figures 2C,D, 3D, 4A–C, 5D, 6A–C, 7D, and 8A–C; *T. brasiliensis*: Figures 9A, 10A, and 11A). Our data highlight a selective expression of Calbindin in some prethalamic and most thalamic derivatives (*M. myotis*: Figures 5C,D, 6A–C, 16E,F, and 17E,F; *T. brasiliensis:* Figure 9A′) and the expression of TH in the substantia nigra pars compacta (SNc) and VTA in the basal plate of all diencephalic neuromeres (see below) in *M. myotis* and *T. brasiliensis* (*M. myotis*: Figures 7D, 8A–C, 15C, 16D, and 17C,D; *T. brasiliensis*: Figures 9A, 10A, and 11A).

The dp3 prosomere in these Chiroptera species limits rostrally with the PHy or hp1 prosomere, a boundary that previously was identified with the caudal border of the catecholaminergic tracts from the SNc to the CPu (ns), but also from VTA to nucleus Acb (vta‐l), and to prefrontal cortex (vta‐c) passing through the PHy (*M. myotis*: Figures 7D and 8A,B, *T. brasiliensis*: Figure 9A). Another observation was that the caudal border of the cp, by coursing through the PHy neuromere, establishes this same boundary between PHy and dp3 (*M. myotis*: Figures 3C,D, 4A, 5D, and 7C,D; *T. brasiliensis:* Figure 9A′). The caudal border of the dp3 alar plate can be identified by the mammillothalamic tract (mth) (Calbindin positive) coursing through the rostral alar plate of dp2 in *M. myotis* and *T. brasiliensis* (*M. myotis*: Figures 6B,C, 13F, 15A,B, 16F, and 17E,F; *T. brasiliensis:* Figure 10A′) (J. L. Ferran et al. 2025; Lucero‐Arteaga et al. 2025). Calbindin staining in these species revealed prethalamic derivatives, such as the reticular nucleus (Rt). This nucleus is clearly positioned caudal to the cp and catecholaminergic (VTA‐l, VTA‐c) tracts and rostral to the mth in these chiropteran species (*M. myotis*: Figures 5D, 6A–C, 16E,F, and 17E,F; *T. brasiliensis:* Figures 9A′ and 10A′). In *M. myotis* and *T. brasiliensis*, the mth originates in the mammillary body. Initially, it runs alongside the mammillotegmental tract (mtg; a pathway to the rostral hindbrain). However, at the basal plate of dp3, the two tracts diverge, and the mth makes a 90‐degree ventrodorsal turn (*M. myotis*: Figures 6B–C and 17E,F; *T. brasiliensis:* Figure 10A′). Subsequently, it enters the alar plate of dp2, ultimately reaching the anterior thalamic nuclei (*M. myotis*: Figures 6B,C and 14E,F; *T. brasiliensis:* Figure 10A′). Although the trajectory of this tract in this species within the alar plate of dp2 resembles that reported in the Mongolian gerbil, its precise trajectory in the basal plate of mice and rats has not yet been clearly determined (J. L. Ferran et al. 2025; Lucero‐Arteaga et al. 2025; L. Puelles et al. 2021). TH‐positive dopaminergic neurons in these species were observed in the SNc and VTA of the dp3, supporting the concept of a multi‐neuromeric developmental origin for these rhombo‐meso‐diencephalic dopaminergic cell groups (*M. myotis*: Figures 7D and 8A–C; *T. brasiliensis:* Figures 9A, 10A, and 11A) (J. L. Ferran et al. 2025; Lucero‐Arteaga et al. 2025).

As observed in rodents, the dp2 prosomere in *M. myotis* and *T. brasiliensis* comprises thalamic and habenular derivatives. This prosomere is characterized by two perpendicular tracts that aid in identifying its rostral and caudal limits. Specifically, the mth, reaching the anterior thalamic nuclei, is positioned in the dp2 alar plate adjacent to the prethalamus (dp3). Conversely, the retroflex tract (rf), extending from the habenular region to the interpeduncular nucleus, is observed near the caudal border of dp2 with dp1 (*M. myotis*: Figures 4B, 6C, 8B,C, 16C–F, and 17C–E; *T. brasiliensis:* Figures 10A,A′ and 11A) (J. L. Ferran et al. 2025; J. L. Ferran and Puelles 2024; Lucero‐Arteaga et al. 2025; L. Puelles et al. 2012; L. Puelles and Rubenstein 2003). Calbindin immunostaining in the selected chiropteran species revealed that virtually all thalamic derivatives were immunoreactive, whereas their habenular counterparts consistently lacked Calbindin expression (*M. myotis*: Figures 5B–D, 6A–C, 16E–F, and 17E–F; *T. brasiliensis:* Figures 9A′ and 10A′). As previously described, dopaminergic (TH‐positive) neurons were identified within the SNc and VTA of the dp2 prosomere in both species (*M. myotis*: Figures 7D, 8A–C, 16D, and 17C,D; *T. brasiliensis:* Figures 9A, 10A, and 11A). A species‐specific distinction, however, was the exclusive observation of a cluster of TH‐positive neurons within the medial habenula (MHb) of *M. myotis*, a feature not detected in *T. brasiliensis* (*M. myotis*: Figures 8C, 11C,E, 14E, and 16C,D; *T. brasiliensis:* Figure 11A,B,D). This feature determines that part of the rf is TH positive in *M. myotis* (*M. myotis*: Figures 8B, 14E,F, 16C,D, and 17C,D).

The dp1 prosomere in rodents is characterized by the pretectal region within its alar plate, with the posterior commissure serving as a key anatomical landmark (J. L. Ferran et al. 2007, 2008, 2009, 2025; J. L. Ferran and Puelles 2024; Lucero‐Arteaga et al. 2025; E. Puelles, Martínez‐de‐la‐Torre, Watson et al. 2012; L. Puelles, Martinez‐de‐la‐Torre, Ferran, et al. 2012). The dp1 prosomere in *M. myotis* and *T. brasiliensis* extends from the caudal border of the rt to the caudal border of the posterior commissure (*M. myotis*: Figures 4B,C, 6B–C, 8B–C, and 17A,C,D; *T. brasiliensis*: Figures 9A,C, 10A,A′,C, and 11A,D,E). The caudal border of the posterior commissure serves as the identifying landmark for the dp1/mp1 (diencephalon‐midbrain) border in vertebrates (Brozko et al. 2022; J. L. Ferran et al. 2007, 2008, 2009, 2025; Lucero‐Arteaga et al. 2025; Merchan et al. 2011; Morona et al. 2011, 2017). In the selected chiropteran species, TH‐positive neurons are observed within the SNc and VTA of the dp1 prosomere. The rf, descending through the basal plate of dp2, separates this dopaminergic diencephalic group rostrally from that located in the dp1 and midbrain (mp1 and mp2) neuromeres caudally (*M. myotis*: Figures 7D and 8A–C, *T. brasiliensis:* Figure 11A,D,E). This observation supports a multineuromeric distribution of the SNc and VTA, extending beyond the diencephalic neuromeres to include the midbrain and rostral hindbrain (see below) (J. L. Ferran et al. 2025; Lucero‐Arteaga et al. 2025).

### 3.3 Midbrain of the *M. myotis* and *T. brasiliensis*

In rodents, the midbrain proneuromeric region comprises two neuromeres. The alar plate of mp1 gives rise to, from rostral to caudal, the tectal gray (TG), SC, and IC. Concurrently, the basal plate of mp1 contains the oculomotor nucleus (III cranial nucleus) and its associated III nerve, alongside the red magnocellular nucleus (RMC) (J. L. Ferran et al. 2025; Lucero‐Arteaga et al. 2025; E. Puelles et al. 2012; L. Puelles and Hidalgo‐Sánchez 2023). Our material identifies in *M. myotis* and *T. brasiliensis* the alar plate derivatives defined as TG, SC, and IC and its basal plate with the 3N and a few fibers that seem to belong to the III nerve (*M. myotis*: Figures 3C,D, 4A–C, 5C,D, 6A–C, 7C,D, 8A–C, and 15A–D; *T. brasiliensis:* Figures 9A,C, 10A,A′,C, and 11A,D,E). A striking aspect observed is the big size of the IC in both species (*M. myotis*: Figures 3C,D, 4A, 5D, 6A, 7C,D, and 8A; *T. brasiliensis*: Figures 9A and 11A) that also seems to be proportionally bigger than in rodents (Figures 9B and 10B). The mp2 which corresponds to the preisthmic derivatives is also observed in the selected Chiroptera species (*M. myotis*: Figures 4B,C, 6A–C, and 8A–C; *T. brasiliensis*: Figures 9A, 10A, and 11A). The caudal boundary of the midbrain with the hindbrain, a boundary between mp2 and r0 neuromeres, can be recognized in *M. myotis* and *T. brasiliensis* by de decussation of the superior cerebellar peduncle and the rostral border of the interpeduncular nucleus, as occurs in rodents (*M. myotis*: Figures 4B,C, 6B,C, and 8A,B; *T. brasiliensis:* Figures 9A, 10A, and 11A) (J. L. Ferran et al. 2025; Lucero‐Arteaga et al. 2025; E. Puelles et al. 2012; L. Puelles and Hidalgo‐Sánchez 2023).

### 3.4 Hindbrain of the *M. myotis* and *T. brasiliensis*

The hindbrain develops as a series of 13 segmental units called rhombomeres (r0–r12), which establishes its fundamental anteroposterior organization. These rhombomeres are grouped into four distinct proneuromeric functional regions. The PrP region is formed by rhombomeres r0, r1 rostral (r1r), and r1 caudal (r1c). Caudal to this, rhombomeres r2, r3, and r4 define the pontine region. The RP region comprises rhombomeres r5 and r6, whereas the most caudal rhombomeres, r7 through r11, constitute the Me region (Figure 2C,D). This precise segmentation is crucial for the proper patterning and differentiation of hindbrain structures (Figure 2C,D) (Nieuwenhuys and Puelles 2016; L. Puelles 2018; L. Puelles and Hidalgo‐Sánchez 2023; Tomas‐Roca et al. 2016; Watson et al. 2017). NeuN immunoreactivity serves as a valuable immunoreaction for discriminating the distinct rhombomeric units r0 to r4 within the hindbrain of bat species, specifically *M. myotis* and *T. brasiliensis* (Figure 4B,C). NeuN (neuronal nuclei) is a widely recognized neuronal nuclear antigen, expressed in the nuclei and perinuclear cytoplasm of most differentiated neurons in the central nervous system. Its consistent expression in postmitotic neuroblasts and mature neurons makes it a robust marker for identifying neuronal populations (Mullen et al. 1992). Specific hindbrain populations, notably the SNc and VTA within the most rostral rhombomere (r0), exhibit TH immunoreactivity. This finding supports the widespread, multineuromeric distribution of these neuronal populations across diencephalic (dp1–dp3), midbrain (mp1–mp2), and rostral hindbrain (r0) neuromeres (*M. myotis*: Figure 8C; *T. brasiliensis:* Figure 11A). In these microchiropteran species, the midbrain–hindbrain boundary (mp2–r0) is identifiable rostral to the decussation of the superior cerebellar peduncle (dscp) (*M. myotis*: Figures 4B,C, 6B,C, and 8A,B; *T. brasiliensis:* Figures 9A, 10A, and 11A). Moreover, the rostral border of the interpeduncular nucleus serves as an additional key anatomical landmark for this boundary (*M. myotis*: Figures 4C and 8B,C) (J. L. Ferran et al. 2025; Lorente‐Canovas et al. 2012; Lucero‐Arteaga et al. 2025; L. Puelles 2018). Compared with the rodent's pontine nucleus, it appears proportionally increased in size in these microbat species (*M. myotis*: Figures 3D, 4B,C, 5D, 6A–C, 7D, and 8A–C; *T. brasiliensis:* Figures 9A, 10A, and 11A). The cerebellar foliation patterns of *M. myotis* and *T. brasiliensis* are similar, which contrasts with the distinct differences observed between microbats and rodents, the latter of which have a more pronounced and complex pattern (*M. myotis*: Figures 4B,C, 6B,C, and 8B,C; *T. brasiliensis:* Figures 9A–D, 10A–D, and 11A). The study highlights rhombencephalic auditory components, specifically the cochlear (Co) nucleus and the superior olive (SO) complex. In both *M. myotis* and *T. brasiliensis*, these structures appear proportionally larger when compared to those in rodents (*M. myotis*: Figures 3D, 5D, 6A, 7D, 8A, and 15E; *T. brasiliensis*: Figure 9A′). Calbindin expression was observed within the Co nucleus of both microchiropteran species (*M. myotis*: Figures 5D and 6A; *T. brasiliensis*: Figure 9A′).

## 4 Discussion

We found that the neuromeric modular organization (i.e., forebrain prosomeres and hindbrain rhombomeres) in *M. myotis* and *T. brasiliensis* is conserved and comparable to that of rats, mice, and Mongolian gerbils. We also identified most of the tracts and anatomical landmarks that define the boundaries and components of these neuromeres (J. L. Ferran et al. 2025; Lucero‐Arteaga et al. 2025; L. Puelles 2018; L. Puelles and Rubenstein 2015). These findings reinforce the concept that specific genetic programs within each neuromeric unit are conserved throughout the vertebrate brain development (ontogeny) and evolution (phylogeny) (J. L. Ferran, Puelles, 2015, 2017, 2025; Lucero‐Arteaga et al. 2025; L. Puelles 2013, 2018; L. Puelles and Hidalgo‐Sánchez 2023; L. Puelles and Rubenstein 1993, 2003, 2015; Tomas‐Roca et al. 2016). However, our study also revealed that *M. myotis* and *T. brasiliensis* exhibit several distinct differences from rodents in their neuromeric derivatives. These microbats have a markedly reduced cc and isocortex and smaller och, III nerve fibers, and cerebellar foliation. On the other hand, they exhibit a proportionally larger anterior and hc, CPu, Acb nucleus, pallial and subpallial amygdala, and enlarged IC, pontine nuclei, Co nucleus, and SO complex. Interestingly, no compact tract for the postcommissural fornix was observed in both *M. myotis* and *T. brasiliensis*. Additional studies will be required to determine if the enlarged CPu and Acb nucleus are functionally or structurally related to regional variations within the SNc and the VTA. Although the enlarged hippocampal formation and amygdala may account for the increased axonal input to, and subsequent enlargement of, the hippocampal and ac (a characteristic observed in marsupials and xenarthrans), the reduced size of the cortex likely results in fewer axons passing through the cc, thereby diminishing its size, similar to the condition found in xenarthrans (Jakob and Onelli 1913; Smith 1897, 1899; Suárez et al. 2018). Collectively, these observations indicate that several changes have occurred over the 85 Mys since the last common ancestor of Laurasiatheria (which includes bats) and Euarchontoglires (the superorder of rodents, primates, and others).

Microchiroptera bats rely on echolocation, a specialized sensory capability that is crucial for defining their ecological niche and executing foraging activities. The specific type of echolocation utilized directly governs the niche and feeding behaviors in species like *M. myotis* and *T. brasiliensis* (Amaral et al. 2023; Norberg and Rayner 1987; Russo et al. 2007; Schnitzler et al. 2003). To achieve this precision, these bats have developed intricate peripheral and central specializations in their auditory system. Central specializations are notably observed across a series of brain regions, including the Co nucleus, SO complex, nucleus of the lateral lemniscus (NLL), IC, auditory thalamus, and auditory cortex (Covey 2005). Microchiroptera bats, when compared to rodents, exhibit a proportional increase in the size of key central auditory structures, specifically the Co nucleus, the SO complex, and the IC. However, a critical need remains for further detailed studies to determine if this volumetric increase is uniform across all anatomical partitions of the Co nucleus (e.g., dorsal vs. ventral Co). Additionally, research must investigate whether differences in the fundamental cellular or molecular composition within these specialized nuclei can mechanistically account for the distinct echolocation behaviors observed between species such as *M. myotis* and *T. brasiliensis*.

Typically, the postcommissural fornix is organized as a compact tract in rodents. However, such anatomical feature is not apparent in the analyzed Microchiroptera species, indicating that the hippocampal projections to the mammillary body and other hypothalamic targets are not organized as a distinct tract in this clade. It is therefore conceivable that the individual axons originating from the hippocampus are diffusely distributed throughout the peduncular and terminal hypothalamic neuromeres, rather than being confined to the rostral border as seen in most mammals. A similar non‐compact organization was observed in the chicken brains’ rf, which follows a dispersed pattern (J. L. Ferran and Puelles 2024). On the other hand, the trajectory of the mammillothalamic tract along the basal plate of dp3 and the alar plate of dp2, reaching the anterior thalamic nuclei, appears to be a common ancestral trait. This is a shared characteristic between the superorders Laurasiatheria (including bats) and Euarchontoglires (including rodents and primates), which diverged approximately 85 Mys ago. Further investigation is needed to assess whether the trajectory of the mammillothalamic tract is also conserved in mice, particularly within the dp2 basal plate (J. L. Ferran et al. 2025; Lucero‐Arteaga et al. 2025; L. Puelles, Martinez‐de‐la‐Torre, Ferran, et al. 2012).

The distribution of TH‐positive processes through the basal plate of the diencephalon‐midbrain‐rostral hindbrain along with the catecholamine tracts is shared between the two microbats, which resembles those described in rodents (mice, rats, and gerbils) and primates (J. L. Ferran et al. 2025; Lucero‐Arteaga et al. 2025). However, our study also revealed a selective TH expression in the MHb of *M. myotis* that was not observed in *T. brasiliensis*. The absent of TH expression in the MHb has been previously reported in other microchiropteran species, including *Cardioderma cor*, *Chaerephon pumilus*, *Coleura afra*, *Hipposideros commersoni*, *Triaenops persicus*, and *Miniopterus schreibersii* (Kruger et al. 2010; Maseko and Manger 2007). Similarly, TH expression in rodents is only observed in the lateral habenula (LHb), whereas it is lacking in the MHb (Figure 10B) (Bilbao et al. 2022; J. L. Ferran et al. 2025; Namboodiri et al. 2016; Wada et al. 2015). On the other hand, TH expression is absent in both the MHb and LHb in the chicken avian brain and in some reptiles like *Pseudopus apodus, Pseudemis scripta elegans*, and *Gekko gecko* (Domínguez et al. 2010; Jiménez et al. 2025; L. Puelles and Medina 1994; Reiner et al. 1994). It is therefore possible that the selective expression in the MHb in *M. myotis* is linked to an evolutionary novelty within this species, whereas its absence is a defining characteristic of a clade involving rodents and bats. Further work is needed to determine the origin and functional role of TH‐positive processes in the MHb.

Collectively, our study revealed that despite the observed differences, the neuromeric organization is conserved within specific compartments, while still allowing for variations in the relative size of their derivatives (L. Puelles 2009; L. Puelles and Rubenstein 2003, 2015). As illustrated here, the prosomeric framework provides a valuable tool for understanding brain development in bats from which specific neuromeric derivatives can be identified as homologous with other species because they arise from the same developmental compartment of vertebrates (J. L. Ferran 2017; L. Puelles and Ferran 2012; L. Puelles and Medina 2002). In addition, delineating the precise boundaries of neuromeres and its derivatives are key steps for determining the extent to which convergent or divergent processes of evolution occurred within the suborder of microbats (Rueda‐Alana et al. 2025).

## Author Contributions

All authors have read and approved the final version of the manuscript. Conceptualization: K. Y. Tseng and J. L. Ferran. Brain preparation: F. Lucero‐Arteaga, A. Abrego‐Alvarez, M. Clauzure, S. Labegorra, V. Heck, A. I. Portu, M. A. Boeris, and M. A. Mondino. Histology and immunochemistry: F. Lucero‐Arteaga, B. Ribeiro Do‐Couto, M. Á. García‐Cabezas, and J. L. Ferran. Data analysis: F. Lucero‐Arteaga, A. Abrego‐Alvarez, M. Clauzure, S. Labegorra, V. Heck, A. I. Portu, M. A. Boeris, M. A. Mondino, B. Ribeiro Do‐Couto, K. Y. Tseng, M. Á. García‐Cabezas, and J. L. Ferran. Writing – original draft preparation: K. Y. Tseng, M. Á. García‐Cabezas, and J. L. Ferran. Writing – review and editing: All authors (F. Lucero‐Arteaga, A. Abrego‐Alvarez, M. Clauzure, S. Labegorra, V. Heck, A. I. Portu, M. A. Boeris, M. A. Mondino, B. Ribeiro Do‐Couto, K. Y. Tseng, M. Á. García‐Cabezas, J. L. Ferran). Funding acquisition: K. Y. Tseng, M. A. Boeris, J. L. Ferran. Supervision: J. L. Ferran.

## Funding

The funding was supported by the Spanish Ministry of Science, Innovation and Universities and European Regional Development Fund (FEDER; PGC2018‐098229‐B‐100 to JLF), the Seneca Foundation‐Science and Technology Agency of the Region of Murcia (21,903/PI/22 to JLF), the Faculty of Veterinary Sciences of the University of La Pampa—Argentine (048‐2023 to JLF and MB), and institutional funds from the University of Illinois Chicago—College of Medicine (KYT).

## Ethics Statement

The original research reported herein was performed according to the protocol approved by the Advisory Committee for the Care and Use of Experimental Animals of the Faculty of Veterinary Sciences of the University of La Pampa for microbats and the Animal Research Ethics Committee (CEEA) of the University of Murcia for rats.

## Conflicts of Interest

The authors declare no conflicts of interest.

## Supporting information



Supplementary figure 1: Western blot.

## Data Availability

The entire collection of images of the histological and immunohistochemical processing are not publicly available due to privacy reasons but are available upon request to the corresponding author.
