# Supplementary figures and images for: Neuromeric Organization of the Microbat Brain: Conserved and Distinct Regional Features
Source: J Comp Neurol. 2026 Feb 17;534(2):e70140. doi: 10.1002/cne.70140 (PMC12914228; doi:10.1002/cne.70140)

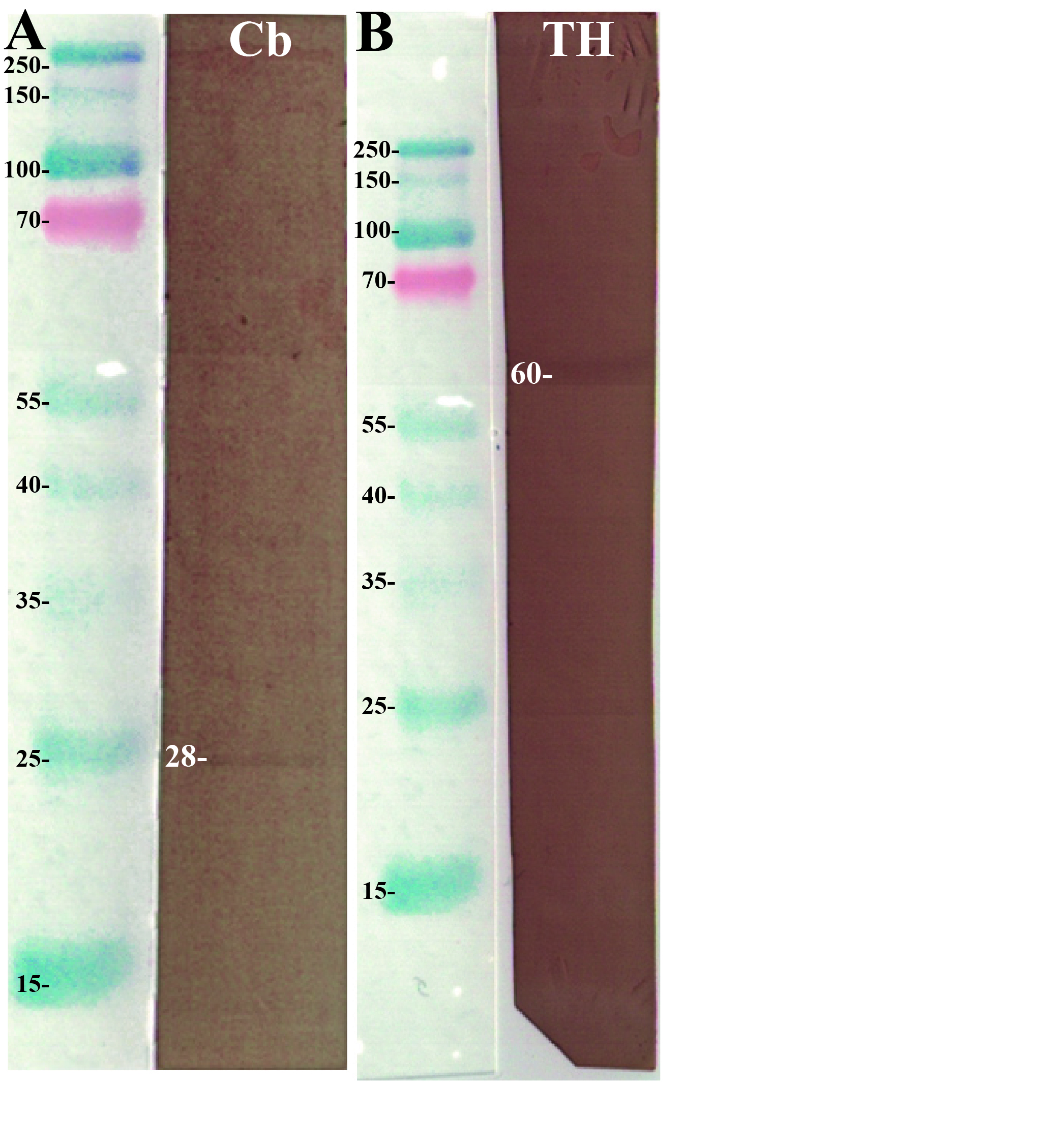

Supplement: Supplementary file 1 — Supplementary figure 1: Western blot. [file CNE-534-e70140-s001.jpg]
